# Homeostasis of DNA Hemi‐Methylation in *Arabidopsis* through Methylation Maintenance, DNA Replication, and Nucleosome Positioning Mechanisms

**DOI:** 10.1002/advs.202505808

**Published:** 2025-07-11

**Authors:** Hengye Chen, Chenhuan Xu

**Affiliations:** ^1^ China National Center for Bioinformation Beijing 100101 China; ^2^ Beijing Institute of Genomics Chinese Academy of Sciences Beijing 100101 China

**Keywords:** CG dyad, DNA methylation, hairpin BS‐seq, hemi‐methylation, maintenance methylation

## Abstract

The DNA methylome in eukaryotes is an equilibrium contributed by maintenance methylation, *de novo* methylation, demethylation, and DNA replication processes. The asymmetric hemi‐methylation status is thought of as a transient intermediate state *en route* to full‐methylation. However, the recent studies in mammalian cells suggest that hemi‐methylated CG dyads in certain regions can be stably maintained and may serve as epigenetic marks. Compared to mammals, plants have more diversified methylomes often including non‐CG dyads. In this work, hairpin BS‐seq is performed to acquire dyad‐resolution methylomes from *Arabidopsis* wildtype and methylation‐deficient mutant lines. Analyses of methylomes across multiple lines reveal how different DNA methyltransferases influence the equilibrium of hemi‐ and full‐methylation in vivo. The results suggest that nucleosomes may protect hemi‐methylated dyads from being further methylated, resulting in relatively higher hemi‐methylation frequencies in nucleosome‐occupied than ‐depleted regions. Adjacent hemi‐methylated dyads tend to have a strong strand‐specificity, indicating that DNA replication is a driving force for the homeostasis of hemi‐methylation. Overall, comprehensive multi‐line and dyad‐resolved DNA methylation maps in *Arabidopsis* is presented to reveal that the homeostasis of DNA hemi‐methylation in plants is achieved through diverse methylation regulatory and chromatin‐related activities.

## Graphical abstract

In *Arabidopsis*, the homeostasis of full‐ and hemi‐methylation is mainly regulated by maintenance methylation. The maintenance methylation at hemi‐methylated dyads is highly efficient but the efficiency differs at euchromatic or heterochromatic regions. Nucleosomes impair the hemi‐ to full‐methylation transition. Lagged methylation maintenance after DNA replication results in strand‐specific hemi‐methylation patterns.

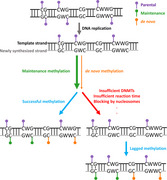

## 1 Introduction

Methylation at the 5′ position of cytosine (5mC) is one of the most common epigenetic modifications in eukaryotes. This modification can alter the binding affinity of DNA to proteins, thereby impacting chromatin structure and gene regulation.^[^ ^1^, ^2^, ^3^, ^4^ ^]^ In mammalian cells, DNA methylation predominantly occurs at CG dinucleotides, whereas, in plants, DNA methylation can occur at cytosines in any sequence context, resulting in a more complex DNA methylome.^[^ ^2^, ^5^, ^6^, ^7^, ^8^ ^]^

In *Arabidopsis thaliana*, the key DNA methyltransferases are METHYLTRANSFERASE 1 (MET1), CHROMOMETHYLASE 2 (CMT2), CMT3, and DOMAINS REARRANGED METHYLTRANSFERASE 1 (DRM1) and DRM2. MET1 is homologous to mammalian DNA methyltransferase 1 (DNMT1), which is responsible for most CG methylation.^[^ ^9^, ^10^, ^11^ ^]^ Previous studies have shown that, similar to DNMT1, MET1 maintains DNA methylation during DNA replication, ensuring the maintenance of CG methylation on newly synthesized DNA strands.^[^ ^2^, ^10^, ^11^, ^12^, ^13^, ^14^, ^15^, ^16^, ^17^ ^]^ However, unlike DNMT1, MET1 lacks the domain that directly interacts with hemi‐methylated CG dyads. Instead, it is recruited to hemi‐methylated CG dyads via its partner, VARIANT IN METHYLATION 1 (VIM1).^[^ ^10^, ^18^, ^19^ ^]^ CMT2 and CMT3 are homologous proteins, but they have distinct substrate sequence specificities. CMT2 catalyzes most CHH methylation (H = A/T/C), while CMT3 is responsible for CHG methylation. In vitro studies of a CMT3 homolog in *Zea mays* suggest that CMT3 recognizes a hemi‐methylated CHG dyad and deposits a methyl group onto the unmethylated cytosine in this CHG dyad.^[^ ^20^, ^21^, ^22^, ^23^, ^24^, ^25^ ^]^ This recognition mechanism is crucial for CMT3's enzymatic activity in vitro, enabling its specific binding to CHG. By contrast, CMT2 lacks an amino acid that interacts with 5mC in CHG, resulting in low catalytic activity at CHG sites.^[^ ^23^, ^26^ ^]^ Interestingly, adding this amino acid to CMT2 restores its ability to efficiently catalyze methylation at CWG dyads (W = A/T).^[^ ^26^ ^]^ Furthermore, the enzymatic activities of both CMT2 and CMT3 are significantly enhanced by H3K9me2, both in vitro and in vivo.^[^ ^22^, ^25^, ^27^ ^]^ This histone mark is primarily deposited by SUPPRESSOR OF VARIEGATION 3–9 HOMOLOG (SUVH) family histone methyltransferases: SUVH4, SUVH5, and SUVH6.^[^ ^28^, ^29^ ^]^ Unlike the DNA methyltransferases mentioned above, DRM1 and DRM2 (DRM1/2) catalyze DNA methylation through the RNA‐directed DNA methylation (RdDM) pathway, enabling the deposition of methyl groups on cytosines in any DNA context.^[^ ^2^, ^20^, ^21^, ^30^ ^]^ This pathway also requires DRM3, a catalytically inactive homolog of DRM1/2.^[^ ^31^ ^]^ DRM1/2 is particularly important for *de novo* methylation in *Arabidopsis*. Previous studies suggest that DRM2 deposits methyl groups more continuously at CHH sites than CMT2, due to its higher processivity.^[^ ^32^ ^]^

The distribution of DNA methylation is well‐studied in *Arabidopsis*. CG, CHG, and CHH methylation are enriched at centromeres and pericentromeres, which contain transposable elements (TEs), often consisting of repetitive sequences, thereby repressing the transcriptional activity of these elements and maintaining genome stability.^[^ ^2^, ^4^, ^5^, ^6^, ^7^, ^10^, ^19^, ^33^, ^34^ ^]^ In addition to its repressive functions, CG methylation in gene bodies promotes gene expression in euchromatic regions.^[^ ^2^ ^]^

Hemi‐methylation and full‐methylation are two interchangeable statuses of cytosine dyads under certain circumstances. The homeostasis of hemi‐methylation and full‐methylation within a DNA methylome is a net consequence of different processes, including DNA replication, maintenance methylation, *de novo* methylation, and demethylation. Studies in mammalian cells suggest that hemi‐methylation can be maintained across multiple cell cycles. Strand‐specific CG hemi‐methylation is found near CTCF motifs and anti‐correlates with the nucleosome positioning.^[^ ^35^, ^36^ ^]^ In plants, the quantity and distribution of hemi‐methylation have not been characterized on a genome‐wide scale. Based on the studies of DNA methyltransferases and methylation maintenance in *Arabidopsis*, we hypothesized that plant DNA methylomes commonly contain hemi‐methylation for four reasons. First, MET1 recognizes hemi‐methylated CG dyads via an indirect mechanism, which may affect the maintenance efficiency.^[^ ^10^ ^]^ Second, the catalytic activity of CMT3 highly relies on H3K9 methylation, therefore in regions with low H3K9 methylation, CMT3 may maintain symmetric CWG methylation inefficiently.^[^ ^22^, ^25^, ^26^, ^37^ ^]^ Third, the CG and CWG methylation frequencies are intermediate in the euchromatic regions, suggesting the presence of hemi‐methylated dyads.^[^ ^7^ ^]^ Lastly, previous studies have found genome‐wide strand bias in plant DNA methylomes, strongly indicating the existence of hemi‐methylation in plants.^[^ ^20^, ^22^, ^38^ ^]^ Furthermore, the diversified DNA methylation context in plants offers a unique opportunity to investigate and compare hemi‐methylation dynamics and mechanisms across CG and non‐CG dyads, which is not feasible in mammals.

In standard whole‐genome bisulfite sequencing (WGBS) analysis, DNA full‐ and hemi‐methylation are indistinguishable, resulting in the potential misinterpretation of methylation statuses at certain genomic loci. To systematically investigate the quantity and function of hemi‐methylation in plants, we performed hairpin bisulfite sequencing (hpBS‐seq) on a wild‐type *Arabidopsis thaliana* line, Col‐0, as well as lines mutated in various components of methylation‐related pathways.^[^ ^39^ ^]^ By resolving hemi‐ and full‐methylation at CG, CWG, and CWWG dyads, we propose that DNA replication is the primary determinant for generating hemi‐methylated dyads. Generally, these hemi‐methylated CG and CWG dyads are efficiently converted to fully methylated ones, but the maintenance methylation in heterochromatic regions is less efficient than that in euchromatic regions. Hemi‐methylated dyads in nucleosomal regions are protected by nucleosomes from further methylation by DNA methyltransferases, while hemi‐methylated CG and CWG dyads in accessible regions are more likely to be specifically recognized and methylated by MET1 and CMT3, respectively. Nucleosomes also play a crucial role in maintaining symmetric methylation at CWG dyads by recruiting CMT3 via H3K9 methylation.

## 2 Results

### 2.1 Hemi‐Methylation is a Ubiquitous Component in Plant DNA Methylome

**Figure 1:**
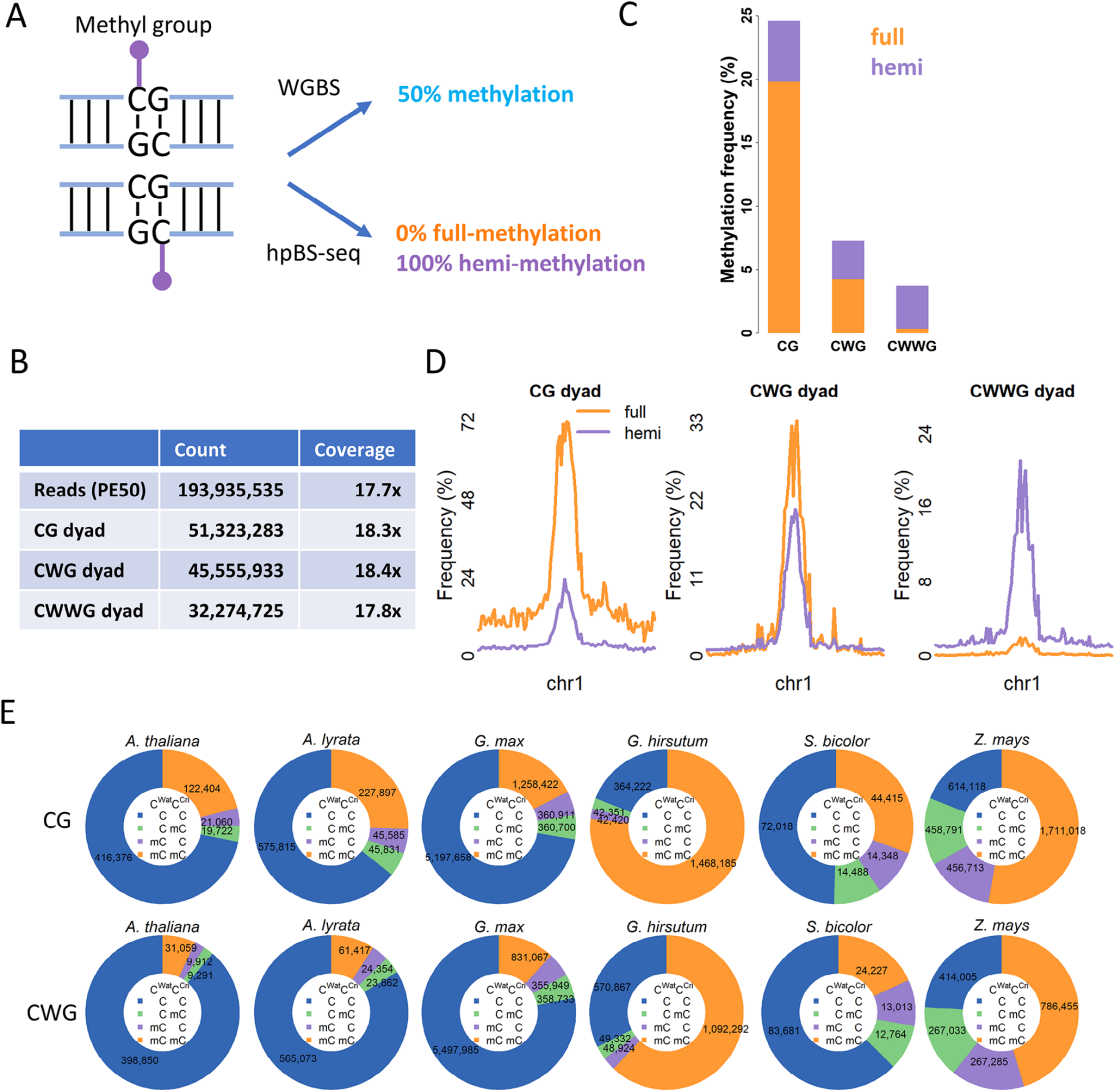
DNA full‐ and hemi‐methylation frequency at different DNA contexts in *Arabidopsis thaliana* and other plant species. A) hpBS‐seq, but not WGBS, can resolve hemi‐methylated dyads. B) The technical statistics of hpBS‐seq dataset in Col‐0. Three biological replicates were included. PE: paired‐end. C) Methylation frequency of CG, CWG, and CWWG dyads. full: full‐methylation. hemi: hemi‐methylation. D) Hemi‐ and full‐methylation frequency of CG, CWG, or CWWG dyads on Chromosome 1 at 300‐kb resolution. E) iSA‐resolved proportions of unmethylated, hemi‐methylated, and fully methylated CG or CWG dyads from WGBS datasets from *A. thaliana*, *A. lyrata, G. max, G. hirsutum, S. bicolor, and Z. mays*. C^Wat^ and C^Cri^ represents the methylation status of the cytosine on Watson and Crick strands, respectively.

We performed hpBS‐seq in *Arabidopsis* wild‐type line Col‐0, to distinguish hemi‐methylated cytosine dyads from fully‐methylated dyads, thereby providing a dyad‐resolved DNA methylome (**Figure** **1**A,B). To test the reliability of the acquired hpBS‐seq datasets, we first compared CG methylomes captured by hpBS‐seq and WGBS in *Arabidopsis*.^[^ ^11^ ^]^ As expected, hpBS‐seq and WGBS display comparable CG methylation frequencies at single base resolution, and hpBS‐seq datasets are highly reproducible between replicates (Figure S1A,B, Supporting Information). Besides CG dyads, methylation at CHG dyads can also be maintained in a symmetric manner. Since the reverse complement of CCG is not in a CHG context, we focused mainly on CWG dyads in this work. CHH context is another target of methyltransferases in *Arabidopsis*. Unlike CG and CWG dyads, CHH dyads do not contain two symmetric cytosines. Instead, CWWG dyads contain two symmetric cytosines, both within CHH context, enabling the study of hemi‐ and full‐methylation at CHH context. We then investigated hemi‐methylation at CG, CWG, and CWWG dyads to gain a comprehensive understanding of the methylation dynamics at different cytosine context (Figure 1C). In *Arabidopsis thaliana*, CG is the most frequently methylated context, with full‐methylation frequencies reaching up to 20%, while hemi‐methylation frequency at CG dyads is ≈4% in average (Figure 1C). Besides CG dyads, CWG dyads are also frequently methylated, but their full‐methylation frequency is ≈3 times lower than that of CG dyads (Figure 1C). Unlike full‐methylation, the hemi‐methylation frequencies at CWG and CG dyads are close, suggesting that the maintenance of symmetric methylation is less efficient at CWG dyads than CG dyads. Full‐methylation frequency at CWWG dyads is lower than at the other two DNA contexts, and hemi‐methylation is the dominant form of CWWG methylation (Figure 1C). This suggests that methylation at CWWG dyads is likely stochastic between two DNA strands and across different cytosines, whereas CG and CWG employ specialized mechanisms for maintaining symmetric methylation between two DNA strands.

Previous WGBS results showed that *Arabidopsis* has a unique distribution of DNA methylation within the genome.^[^ ^7^, ^11^ ^]^ Consistent with these findings, our hpBS‐seq data revealed that methylation is enriched at pericentromeres, while other regions exhibit very low methylation frequencies, particularly at CWG and CWWG contexts (Figure 1D; Figure S1C–E, Supporting Information). As expected, hemi‐ and full‐methylation in the same DNA context are highly correlated because both of them are generated by the same enzymes and can be converted between each other (Figure S1F, Supporting Information). The methylation frequencies in different DNA contexts are also positively correlated due to the high 5mC abundance in the pericentromeric regions (Figure 1D; Figure S1C,E, Supporting Information).

In addition to the heterochromatic pericentromeric regions, CG methylation is also enriched within gene bodies.^[^ ^7^ ^]^ To further characterize the distribution of full‐ and hemi‐methylation, we quantified their frequencies across genes stratified by high, moderate, and low transcriptional activity (Figure S1G,H, Supporting Information).^[^ ^5^ ^]^ The analysis revealed that CG full‐methylation is more enriched in gene bodies of highly expressed genes than those of poorly expressed ones, whereas CG hemi‐methylation frequency of highly expressed genes is generally lower than that of unexpressed ones. This finding deviates from the general positive correlation observed between full‐ and hemi‐methylation, implying that hemi‐methylated dyads may be more efficiently converted to fully methylated states, or the fully methylated dyads may be subject to lower turnover rates, in highly transcribed regions than regions with low transcriptional activity.

Since plants have well‐conserved DNA methyltransferases across species,^[^ ^23^ ^]^ we hypothesized that these plants may share similar methylation patterns. To determine whether hemi‐methylation is a ubiquitous feature in plants, we analyzed hemi‐methylation in several other plant species, including *A. lyrata*, *G. max, G. hirsutum*, *S. bicolor*, and *Z. mays*. Because hpBS‐seq data for these plants were unavailable, we retrieved dyad‐resolved methylation information from WGBS data using a method named in silico strand annealing (iSA),^[^ ^40^, ^41^, ^42^, ^43^ ^]^ and found that these species also exhibit relatively high levels of hemi‐methylation (Figure 1E), indicating that hemi‐methylation is a conserved phenomenon in plants.

In summary, our hpBS‐seq results presented a dyad‐resolution methylome at CG, CWG, and CWWG contexts in *Arabidopsis thaliana*. The data shows that hemi‐methylation occurs in all DNA contexts but varies in level, suggestive of distinct methylation dynamics across DNA contexts. Beyond *Arabidopsis thaliana*, multiple other plant species also exhibit relatively high levels of hemi‐methylation at CG and CWG dyads, suggesting that hemi‐methylation is a ubiquitous component in DNA methylomes across the plant kingdom.

### 2.2 Homeostasis of Full‐ and Hemi‐Methylation is Achieved by Overlapping and Distinct Mechanisms

**Figure 2:**
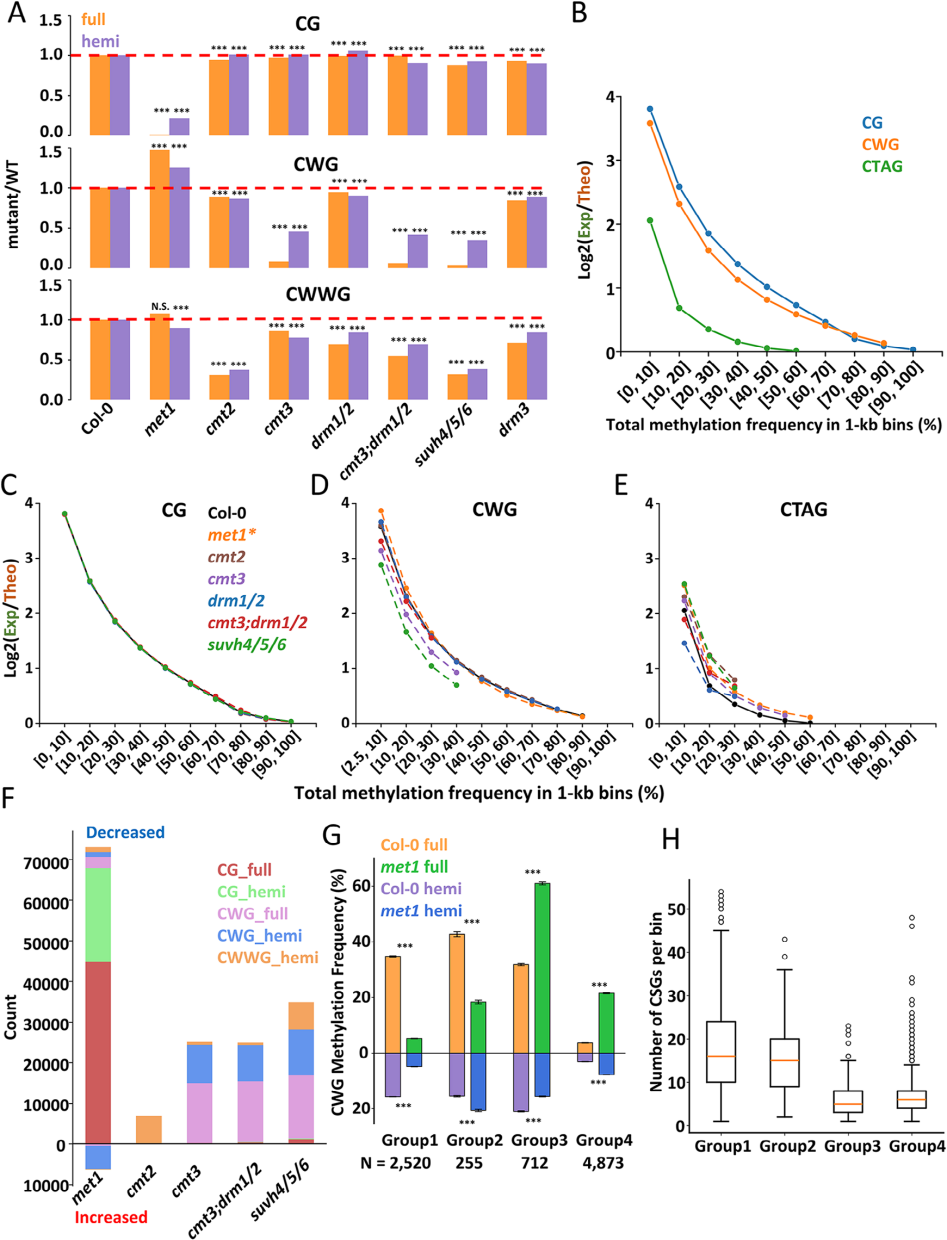
Homeostasis of full‐ and hemi‐methylation is co‐regulated by multiple enzymes. A) Changes of hemi‐ and full‐methylation frequency at CG, CWG, and CWWG dyads in different mutant lines. B) This panel showed the stochasticity of methylation maintenance at CG, CWG, and CTAG dyads. The *y*‐axis represents log2 values of the experimental/theoretical methylation ratio. Large y value represents low stochasticity. y = 0 is indicated by the red dash line. The *x*‐axis represents the range of methylation frequency in each bin. CG, CWG, and CTAG were shown in blue, orange, and green. C–E) The stochasticity of methylation at CG (C), CWG (D), and CTAG (E) dyads in mutant lines, respectively. The *x*‐ and *y*‐axis are same as in (B). Most curves overlap with the WT Col‐0 curve in the CG panel. ^*^: The *met1* mutant result was not shown in the CG panel. F) The numbers of different types of DMRs in mutant lines. Bars on top and bottom of y = 0 represent DMRs with decreased and increased methylation frequencies, respectively. G) The full‐ (top) and hemi‐methylation (bottom) frequencies of Groups 1–4 DMRs in Col‐0 and *met1* mutant lines. H) Numbers of CSG context in Groups 1–4 fDMRs. The box plot shows the middle 50% of numbers. Student's t test was used to calculate the significance *P*‐value.

Since DNA methylation in *Arabidopsis* is regulated by multiple pathways, we performed hpBS‐seq in lines with various mutations in methylation‐related genes to further dissect methylation dynamics (**Figure** **2**A and **Table** **1**; Figure S2A, Supporting Information). Because our samples varied in sequencing depth, we assessed the accuracy of our results by comparing methylation levels across Col‐0 datasets with different coverages. In brief, we calculated the ratio of methylation levels at 2.5x, 5x, 10x, or 15x coverage in 300‐, 50‐, 10‐, 1‐kb, or dyad resolution. Our results suggest that 2.5x coverage is already sufficient for 1‐kb resolution in CG, CWG, and CWWG contexts. However, to obtain reliable dyad‐resolution methylomes, coverage exceeding 15x is recommended (Figure S2B, Supporting Information). Consistent with previous studies, the deletion of MET1 (*met1*), CMT3 (*cmt3*), or CMT2 (*cmt2*) caused significant changes in CG, CWG, or CWWG methylation frequencies, respectively.^[^ ^2^, ^7^, ^9^, ^11^, ^24^, ^34^, ^44^ ^]^ The loss of both DRM1 and DRM2 (*drm1/2*) slightly reduced genome‐wide CWG and CWWG methylation frequencies. Since DRM3 is required by RdDM, the *drm3* mutant exhibited a methylome similar to that of the *drm1/2* mutant.^[^ ^31^ ^]^ In addition to the DNA methyltransferases mentioned above, the loss of SUVH4, SUVH5, and SUVH6 (*suvh4/5/6*) also influenced DNA methylation at CWG and CWWG dyads (Figure 2A; Figure S2A, Supporting Information). From these results, we observed that the deletion of CMT2, DRM1/2, or DRM3 induced comparable fold changes in hemi‐ and full‐methylation frequencies. However, the deletion of MET1, CMT3, or SUVH4/5/6 almost eliminated full‐methylation at CG or CWG dyads, while hemi‐methylation was reduced only by two‐ to five‐fold (Figure 2A). This indicates that MET1 is crucial for maintaining symmetric methylation at CG dyads, while CMT3 and SUVH4/5/6 are important for maintenance methylation at CWG dyads in *Arabidopsis* genome.

**Table 1:** Technical statistics of hpBS‐seq datasets.

Line	Total^a)^	Mapped^b)^		CG dyads		CWG dyads		CWWG dyads	
	Count [M^c)^]	Count [M]	Cov^d)^	Count [M]	Cov	Count [M]	Cov	Count [M]	Cov
Col‐0	194	43	17.7x	51	18.3x	45	18.4x	32	17.8x
*met1*	101	26	10.7x	31	11.2x	27	11.3x	19	10.8x
*cmt2*	152	37	15.0x	43	15.4x	38	15.5x	27	14.9x
*cmt3*	252	35	14.3x	42	15.0x	37	15.0x	26	14.4x
*drm1/2*	18	6	2.6x	7	2.6x	7	2.7x	5	2.6x
*cmt3;drm1/2*	74	9	3.8x	10	3.6x	9	3.7x	7	3.6x
*suvh4/5/6*	316	31	12.4x	34	12.3x	30	12.4x	22	12.2x
*drm3*	221	17	6.9x	18	6.7x	16	6.8x	12	6.7x
*ddm1*	33	6	2.5x	7	2.3x	7	2.3x	5	2.3x

^a)^ Total read count for each line;

^b)^ Reads that can be mapped and paired to the reference genome;

^c)^ M: million;

^d)^ The mean coverage at single‐base‐pair or dyad level.

Since MET1 and CMT3 prefer hemi‐methylated substrates in vitro,^[^ ^22^, ^25^, ^37^ ^]^ we propose that in *Arabidopsis*, MET1 and CMT3 efficiently convert hemi‐methylated dyads into fully‐methylated dyads. By contrast, methylation at CWWG dyads appears to be deposited more stochastically, resulting in low levels of full‐methylation. To test this hypothesis, we assumed that all cytosines are stochastically methylated and calculated the theoretical full‐methylation frequencies based on the overall methylation frequency of 1‐kb bins. To minimize the interference of uneven methylation distribution across different bins (Figure 1C–E), we grouped the bins by their overall methylation frequencies and compared theoretical and observed full‐methylation frequencies for each group (Figure 2B; Figure S2C, Supporting Information). To simplify terminology, we defined the ratio of experimental to theoretical full‐methylation frequencies as the “Exp/Theo” ratio to represent the deflection from the stochasticity model. Because CHH methylation is context‐dependent,^[^ ^7^ ^]^ we focused on the palindromic sequence CTAG, which exhibits the strongest methylation frequencies among all CWWG contexts. In the WT Col‐0 line, the Exp/Theo ratio at CTAG closely matched the stochastic model except for the most poorly methylated bins, whereas CWG and CG dyads exhibited much higher full‐methylation frequencies than predicted by the stochastic model (Figure 2B), suggesting the involvement of specialized maintenance mechanisms.

To understand the role of DNA methyltransferases in maintaining symmetric methylation in each DNA context, we calculated the Exp/Theo ratio for CG, CWG, and CWWG in various DNMT deletion lines. For the analysis of CG dyads, we excluded the *met1* line due to its extremely low CG methylation frequency. The deletion of most factors did not significantly affect the Exp/Theo ratio at CG dyads, suggesting that the symmetry of CG methylation is mainly determined by MET1 (Figure 2C). For the methylation at CWG dyads, deletion of CMT3 reduced the Exp/Theo ratio, but it remained much higher than the stochastic model (Figure 2D). To rule out the influence of DRM1/2, we analyzed the Exp/Theo ratio in the *cmt3;drm1/2* triple mutant, but did not observe a further decrease of the Exp/Theo ratio. Previous in vitro studies showed that CMT2 retains weak activity at CWG dyads,^[^ ^26^ ^]^ suggesting that CMT2 is responsible for the remaining CWG methylation frequencies in the *cmt3;drm1/2* mutant. Furthermore, CMT2 seems to prefer hemi‐methylated CWGs as its substrate.^[^ ^26^ ^]^ Therefore, the Exp/Theo ratio remains high in the *cmt3;drm1/2* mutant. At CTAG dyads, deletion of MET1 increased the Exp/Theo ratio without significantly altering overall CWWG methylation frequencies, likely through indirect effects on other DNA methyltransferases (Figure 2E). Deletion of CMT2 or CMT3 reduced CWWG methylation frequencies and increased the Exp/Theo ratio at CTAG dyads (Figure 2A,E), indicating that these enzymes may stochastically deposit methyl groups at CWWG dyads. Conversely, DRM1/2 deletion reduced both CWWG methylation frequencies and the Exp/Theo ratio, suggesting that DRM1/2 can promote symmetric methylation at CWWG dyads (Figure 2A,E). Because DRM1/2 associates with the transcription machinery in a siRNA‐dependent manner, we propose that they may have a long dwell time at siRNA‐targeted loci, thereby increasing the probability of modifying both cytosines in a dyad. Besides DNA methyltransferases, SUVH4/5/6 strongly influenced the Exp/Theo ratios. Changes caused by SUVH4/5/6 deletion were similar to those observed with CMT2 or CMT3 deletions, indicating that SUVH4/5/6 primarily affects methylation through modulating CMT2 and CMT3 activity. This is consistent with studies showing that H3K9 methylation enhances the catalytic activity of CMT2 and CMT3.^[^ ^22^, ^25^, ^28^, ^29^, ^37^, ^45^ ^]^

Because the methylation frequencies at pericentromeres are much higher than other regions, to check whether different regions have distinct methylation changes, we calculated the fold changes of hemi‐ and full‐methylation frequencies across Chromosome 1 at 300‐kb resolution (Figure S2D–F, Supporting Information). Our data revealed that upon the deletion of MET1, CMT3, or CMT2, the methylation frequencies show larger changes at CG, CWG, or CWWG dyads within the pericentromeric regions than euchromatin regions, respectively (Figure S2D–F, Supporting Information). The differences between the fold changes of hemi‐ and full‐methylation are relatively consistent across the entire chromosome. The effect of SUVH4/5/6 deletion mimicked that of CMT3 and CMT2 deletion at CWG and CWWG dyads, respectively (Figure S2D,E, Supporting Information). By contrast, DRM1/2 primarily affected methylation in euchromatic regions, with its deletion exerting a stronger effect on full‐methylation than on hemi‐methylation (Figure S2E,F, Supporting Information). The deletion of MET1 specifically enhanced CWG methylation in non‐pericentromeric regions, though this effect may be indirect.^[^ ^46^ ^]^

To further understand the dynamics of full‐ and hemi‐methylation in mutant lines, we identified differentially methylated regions (DMRs) for both full‐methylation (fDMRs) and hemi‐methylation (hDMRs) at 1‐kb resolution (Figure 2F). Consistent with the changes in overall methylation, DMRs of each sequence context mainly exist in lines with the corresponding mutant (Figure 2F). We then clustered CG, CWG, and CWWG DMRs into four groups based on the changes in full‐ and hemi‐methylation frequencies: Group 1: both decreased; Group 2: full‐methylation decreased and hemi‐methylation increased; Group 3: full‐methylation increased and hemi‐methylation decreased; and Group 4: both increased. As expected, most fDMRs and hDMRs belong to Group 1 since the deletion of a DNA methyltransferase should decrease both full‐ and hemi‐methylation on its target DNA context. In comparison, Groups 2–4 DMRs are barely observed, except for CWG methylation in the *met1* mutant. To understand why MET1 deletion resulted in paradoxical effects on CWG methylation, we examined the methylation frequencies and DNA sequence contexts of these DMRs. In the WT Col‐0 line, Groups 1–3 DMRs exhibit similar CWG methylation frequencies, while Group 4 DMRs show much lower methylation frequencies compared to the other groups (Figure 2G). Upon the loss of MET1, Groups 1 and 4 DMRs display large fold‐changes in both full‐ and hemi‐methylation. Group 2 DMRs exhibit a two‐fold decrease in full‐methylation and a slight increase in hemi‐methylation, suggesting that the conversion from hemi‐ to full‐methylation is hindered by MET1 deletion. In contrast, the overall methylation frequencies of Group 3 DMRs increase to a high level, indicating that the decrease in hemi‐methylation is due to very high maintenance methylation efficiency. Since CWG methylation highly correlates with H3K9me2, we examined the enrichment of H3K9me2 for each group. Our results showed that some Group 3 and most Group 4 DMRs exhibit increased H3K9me2 enrichment (Figure S2G, Supporting Information). Previous work revealed that MET1 deletion down‐regulates the expression of a histone demethylase IBM1, thus Groups 3 and 4 DMRs may be targets of IBM1.^[^ ^46^ ^]^ By contrast, H3K9me2 enrichment of Groups 1 and 2 DMRs significantly decreased, indicating that the H3K9me2 in these regions may depend on CG methylation. Although MET1 does not directly influence CWG methylation, it can affect methylation at CCG, whose reverse complementary sequence is CGG. To access the influence of CGG methylation on CWG methylation, we calculated the number of CSG (S = C/G) within Groups 1–4 DMRs. Our data showed that Groups 1 and 2 DMRs have 10–20 CSGs per DMR, while Groups 3 and 4 contain only ≈5 CSGs per DMR (Figure 2H). This suggests that MET1 may influence CWG methylation and H3K9me2 by increasing CGG methylation. Therefore, MET1 deletion could lead to opposing effects on CWG methylation through two distinct pathways. Since most of these DMRs overlap with TEs, we investigated whether changes in CWG methylation are associated with specific TE types. Our results showed that the Group 1 DMRs contain more MuDR elements and fewer Helitrons compared to random genomic bins with methylation frequencies are above 10% (Figure S2H, Supporting Information). Compared to Group 1, Groups 2–4 are enriched for the long TEs. Moreover, Groups 3 and 4 contain more En/Spm and Copia. These results suggest that the differential changes in CWG methylation frequencies may relate to the regulation of methylation across different TE families.

Taken together, our data suggest that MET1 efficiently maintains the symmetric methylation status of CG dyads and mildly influences hemi‐methylation frequencies at CWG dyads. CMT2 and CMT3 actively maintain symmetric methylation at CWG dyads and stochastically methylate cytosines at CWWG dyads. Additionally, DRM1/2 deposits methyl groups symmetrically at a small fraction of CWWG dyads, but this symmetry likely arises from prolonged dwell time at specific loci rather than specific recognition of hemi‐methylated dyads. Besides directly modulating methylation at target sequence context, MET1 may also influence CWG methylation through its effects on H3K9 methylation and CGG methylation. SUVH4/5/6 influences methylation at CWG and CWWG dyads by enhancing the enzymatic activity of CMT2 and CMT3.

### 2.3 Heterochromatin and Euchromatin have Distinct Methylation Maintenance Efficiencies

**Figure 3:**
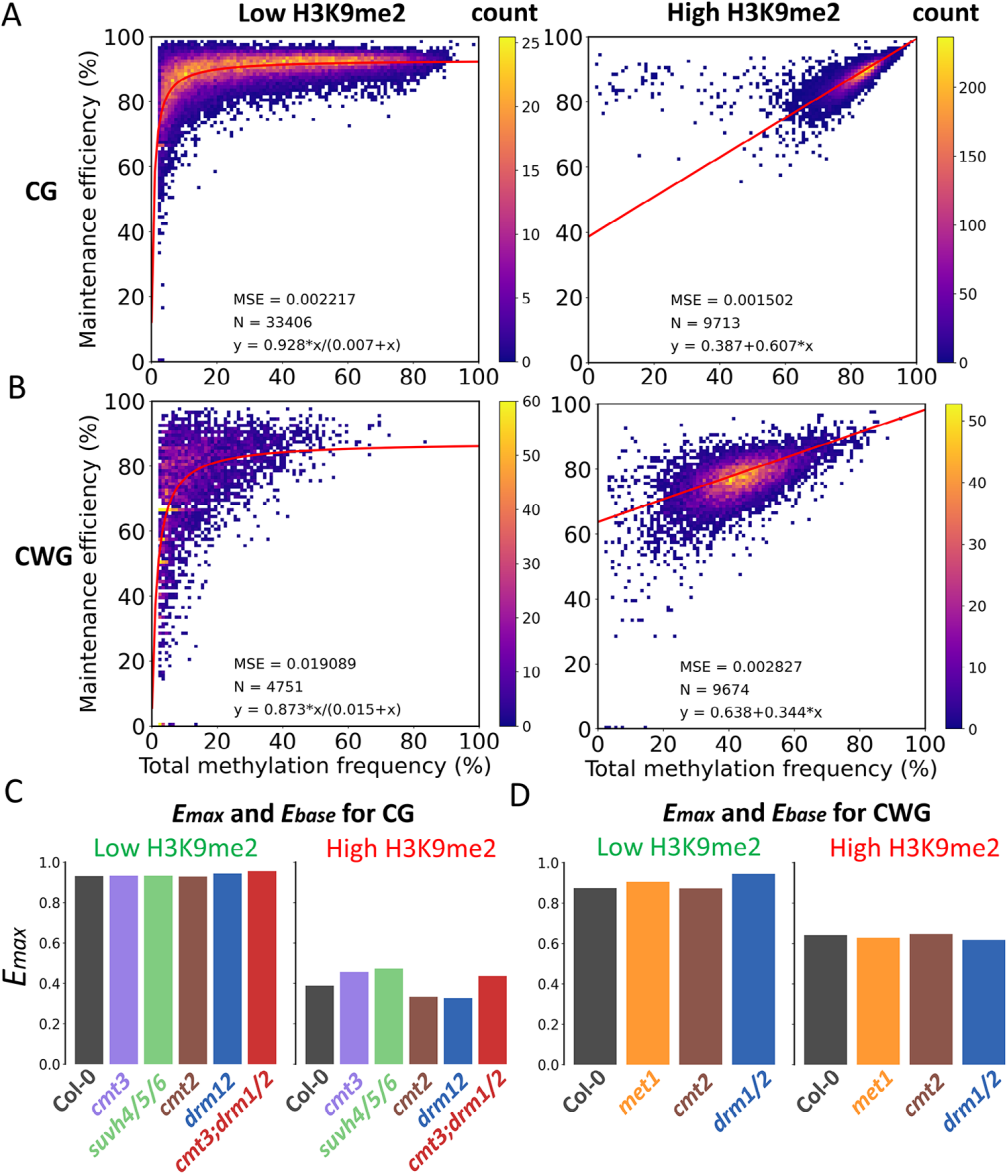
Differential maintenance methylation efficiency at H3K9me2‐enriched and ‐depleted regions. A,B) The maintenance efficiencies of symmetric methylation at CG (A) and CWG (B) dyads are plotted versus the total methylation frequency at 1‐kb resolution. The bins in H3K9me2‐depleted (left) and ‐enriched (right) regions are separately plotted. MSE: the mean square distance. N: the count of bins. The fitted equation is at the bottom of the plot, and the red line is the fitted curve. C,D) The *E_max_* (H3K9me2‐depleted) and *E_base_* (H3K9me2‐enriched) for CG and CWG methylation.

During DNA replication, unmethylated cytosines were incorporated into newly synthesized daughter DNA strands, resulting in double‐strand DNA molecules where cytosine dyads are either hemi‐methylated or unmethylated. Thereby, the efficient maintenance of methylation is important to make sure the methylation information is not lost after a few cell‐cycles. To further dissect the maintenance dynamics of DNA methylation at CG, CWG, and CTAG dyads, we examined the correlation between the maintenance efficiency and the total methylation frequencies (*P_total_*) of each bin (Figure S3A, Supporting Information). The maintenance efficiency is defined as the ratio of full‐methylation frequency (*P_me_*) to total methylation frequency. Since all methylated cytosines are evenly distributed into two daughter cells, the total methylation frequency could be treated as the initial hemi‐methylation frequency after DNA replication. Interestingly, we found that for CG and CWG dyads, the bins could be clustered into two distinct genomic portions: one consisting of bins with various methylation frequencies and the other one primarily comprising highly methylated bins (Figure S3A, Supporting Information). This suggests that the maintenance of symmetric methylation is regulated in two different ways. The major difference between these two portions is that the more highly methylated portion is enriched for H3K9 methylation, a key marker of heterochromatin. Thereby, to better understand the maintenance dynamics of DNA methylation in these two distinct genomic portions, we separately plotted the bins with the lowest and highest H3K9me2 enrichment (**Figure** **3**A,B).

For CG methylation in H3K9me2‐depleted regions, the data points fit a curve similar to the Michaelis–Menten curve, in which the methylation efficiency increases with the total methylation frequency and then reaches a plateau (Figure 3A). This suggests that the maintenance of symmetric CG methylation in H3K9me2‐depleted bins operates in a way similar to an enzymatic reaction in which hemi‐methylated CG dyads function as the substrates. Thus, based on the Michaelis–Menten equation:

(1) $$v_{0}=\frac{V_{max}\left[S\right]}{k_{m}+\left[S\right]}$$

we deduced an equation:

(2) $$\frac{P_{me}}{P_{total}}=\frac{E_{max}\times P_{total}}{k_{m}+P_{total}}$$

where *E_max_* represents the maximum maintenance efficiency, and *k_m_* denotes the affinity between MET1 and hemi‐methylated CG dyads. This model works well for CG methylation in H3K9me2‐depleted regions, and *E_max_* and *k_m_* are 0.928 and 0.007, respectively, indicating highly efficient CG maintenance methylation and high affinity between MET1 and hemi‐methylated CG dyads (Figure 3A). However, in H3K9me2‐enriched regions, the symmetric methylation efficiency does not reach a plateau and cannot be fitted to this equation. Instead, we found the data could be fitted into the equation shown below:

(3) $$\frac{P_{me}}{P_{total}}=E_{base}+k_{p}\times P_{total}$$

In this equation*, E_base_* is a constant representing the basal methylation maintenance efficiency that is independent of *P_total​_*. *k_p_* is a constant representing the correlation between maintenance efficiency and *P_total​_*. For CG methylation in H3K9me2‐enriched regions, *E_base_* and *k_p_* are 0.387 and 0.607, respectively, suggesting that the maintenance efficiency highly correlates with the total methylation frequencies (Figure 3A). This correlation may depend on the local concentration and activity of MET1.

Similar to CG methylation, CWG methylation in these two portions of bins can also be fitted to our models (Figure 3B). In H3K9me2‐depleted regions, *E_max_* and *k_m_* values are 0.873 and 0.015, revealing that CWG maintenance methylation by CMT3 is less efficient than CG maintenance methylation by MET1. By contrast, in H3K9me2‐enriched regions, *E_base_* and *k_p_* values are 0.638 and 0.344, respectively, suggesting that the CWG methylation is more effectively maintained than CG methylation when H3K9me2 is present. This is consistent with the fact in which H3K9me2 enhances the enzymatic activity of CMT3.^[^ ^22^, ^27^ ^]^ For CTAG dyads, we focused only on the H3K9me2‐enriched bins due to the extremely low methylation frequencies in the other bins. We found that the data did not fit our model, suggesting that the symmetric CTAG methylation is not specifically maintained in the same manner as either methylation at CG or CWG dyads (Figure S3A, Supporting Information). In addition, the fitted equation closely approximates to:

(4) $$\frac{P_{me}}{P_{total}}=P_{total}$$

which is consistent with the stochastic model we proposed above. This indicates that the symmetrically methylated cytosines in a CTAG dyad are more likely to result from two separate *de novo* methylation reactions.

We then examined how the deletion of DNA methyltransferases and SUVH4/5/6 influence the efficiency of methylation maintenance. Given that the CTAG methylation is not well‐fitted, we focused only on CG and CWG methylation. For CG methylation in H3K9me2‐depleted regions, the deletion of most factors has no significant effect (*E_max_* change < 5%), except for MET1 (Figure 3C). Since CG methylation is nearly completely lost upon MET1 deletion, we will not discuss it here. In contrast, for CG methylation in H3K9me2‐enriched regions, all factors could influence the CG methylation, since they could directly or indirectly affect H3K9 methylation and chromatin states. For CWG methylation, the deletion of CMT3 and SUVH4/5/6 almost eliminated full‐methylation throughout the genome (Figure 2A,B, Supporting Information). Therefore, in *cmt3*, *cmt3;drm1/2*, and *suvh4/5/6* mutants, the accuracy of curve fitting was decreased by the loss of DNA methylation and H3K9 methylation. But significant maintenance methylation was still observed upon the deletion of CMT3 and SUVH4/5/6 (Figure S3B–D, Supporting Information), suggesting that the symmetric CWG methylation could be maintained by CMT2 in a less efficient manner. MET1 deletion promotes the maintenance of symmetric CWG methylation, consistent with the up‐regulation of CWG methylation upon MET1 deletion (Figures 2A and 3D).^[^ ^46^ ^]^

In summary, we developed a model for the maintenance of symmetric methylation in *Arabidopsis*. In H3K9me2‐depleted euchromatic regions, symmetric methylation at CG and CWG dyads is efficiently maintained at hemi‐methylated dyads after DNA replication, and the maintenance efficiency is relatively constant in highly methylated bins. By contrast, in H3K9me2‐enriched heterochromatic regions, the maintenance efficiency of symmetric methylation is less efficient and positively correlates with the methylation frequency. The enzymatic activities of MET1 and CMT3 determine the maintenance efficiency at hemi‐methylated CG and CWG dyads, respectively, while other DNA methyltransferases and SUVH4/5/6 could also affect the methylation efficiency by altering chromatin environments or catalyzing *de novo* methylation.

### 2.4 CG, CWG, and CWWG Methylation are Differentially Affected by Nucleosomes

**Figure 4:**
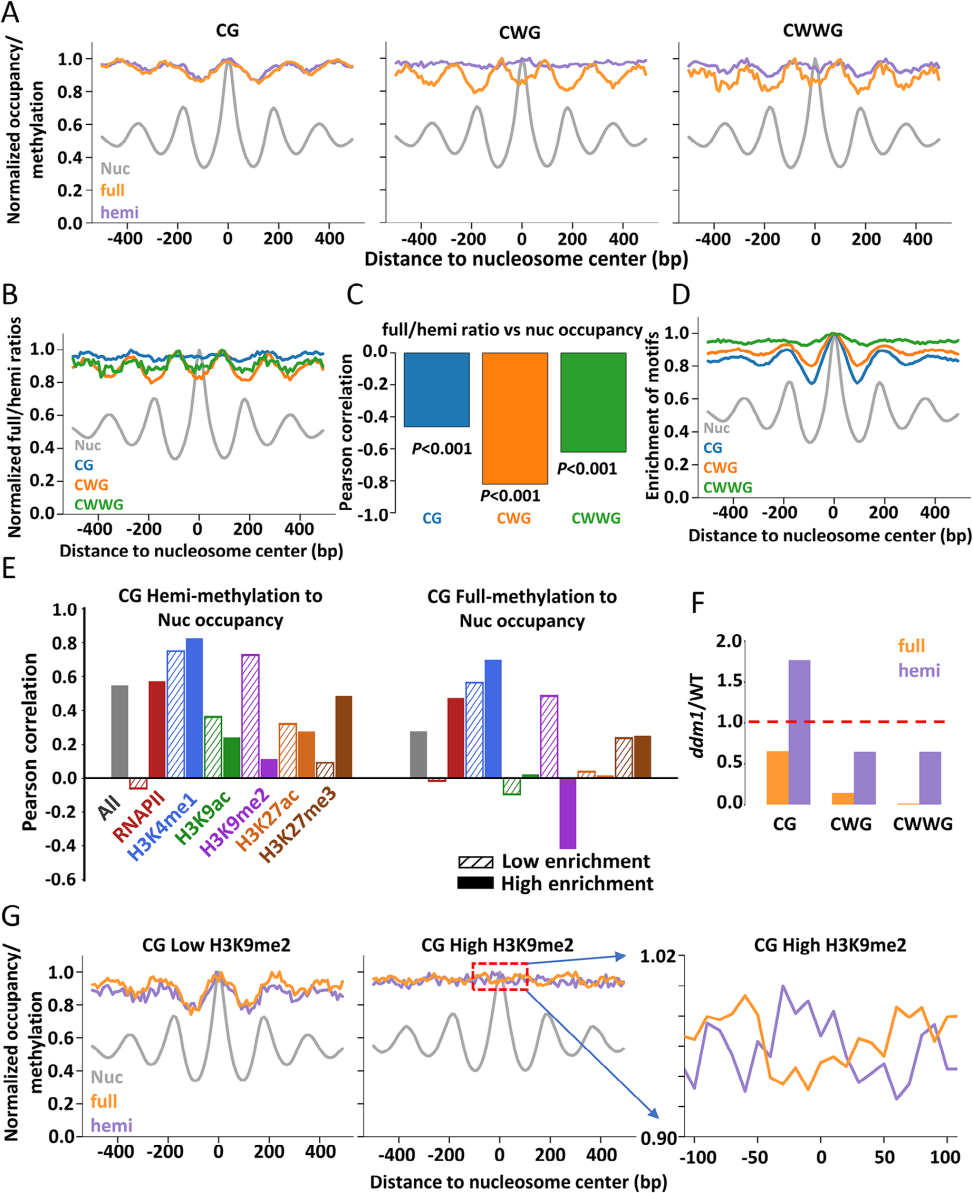
CG, CWG, and CWWG methylation maintenance is interfered by nucleosomes. A) Normalized nucleosome occupancy level (gray), hemi‐methylation (purple), and full‐methylation (orange) frequency near well‐positioned nucleosomes. The highest value in each line was normalized to 1. B) Normalized full/hemi ratios at CG, CWG, and CWWG dyads were plotted over nucleosome occupancy at well‐positioned nucleosomes. C) Pearson correlation coefficient between full/hemi ratio and nucleosome occupancy near well‐positioned nucleosomes. D) The enrichment of CG, CWG, and CWWG sequences near well‐positioned nucleosomes. E) Pearson correlation coefficient between CG methylation frequency and nucleosome occupancy level near well‐positioned nucleosomes in regions with different epigenetic marks indicated by distinct colors. The gray bar represents the genome‐wide correlation. Bars filled by hatches or solid colors represent correlation in regions with low or high enrichment of target marks, respectively. F) Fold‐change of methylation frequencies in *ddm1* mutant. G) Normalized nucleosome occupancy (gray), CG hemi‐methylation (purple), and CG full‐methylation (orange) levels near well‐positioned nucleosomes in H3K9me2‐depleted or ‐enriched regions in *ddm1* mutant. The right panel shows a zoomed view of the area within the red box in the middle panel.

Nucleosome occupancy is a well‐known factor correlated with DNA methylation and transcription factor binding, and periodic methylation patterns have been observed at regions showing phased nucleosomal arrays when the nucleosomes are spaced at regular intervals.^[^ ^22^, ^37^, ^47^, ^48^, ^49^, ^50^, ^51^, ^52^, ^53^, ^54^ ^]^ Since nucleosomes partially protect the DNA surface, we hypothesized that they could influence the homeostasis of hemi‐methylation. To examine how DNA hemi‐methylation is affected by nucleosomes, we calculated hemi‐ and full‐methylation frequencies for CG, CWG, and CWWG dyads near well‐positioned nucleosomes in the *Arabidopsis* genome (**Figure** **4**A).^[^ ^51^ ^]^

Consistent with previous in vivo studies, we found that CG methylation correlates with nucleosome positioning (Figure 4A).^[^ ^47^, ^51^ ^]^ Both hemi‐ and full‐methylation were mildly enriched in nucleosome‐occupied regions (Figure 4A), but the ratio of full‐ to hemi‐methylation was anti‐correlated with nucleosome occupancy near well‐positioned nucleosomes (Figure 4B,C), suggesting that the transition between these two methylation states may be influenced by nucleosome positioning. Additionally, nucleosomal regions exhibited higher CG sequence content compared to linker regions, indicating that sequence context and DNA methylation may influence nucleosome positioning (Figure 4D). In contrast, CWG hemi‐ and full‐methylation displayed distinct patterns near well‐positioned nucleosomes. Specifically, hemi‐methylation did not show a significant correlation with nucleosome positioning, while full‐methylation was anti‐correlated with nucleosome occupancy (Figure 4A–C). This suggests that nucleosomes may obstruct the maintenance of symmetric methylation of CWG dyads. Similarly, CWWG methylation was more enriched in linker regions, and a comparable pattern was observed for both hemi‐ and full‐methylation (Figure 4A). These results further support the notion that nucleosomes impair the deposition of methylation. Similar to the CG context, the CWG and CWWG contexts are also enriched in nucleosome‐occupied regions (Figure 4D), suggesting that nucleosomes may prefer regions with high cytosine content.

As we discussed above, symmetric DNA methylation was differentially maintained in euchromatic and heterochromatic regions. Moreover, a previous work suggests that the correlation between DNA methylation and nucleosome positioning is region‐specific.^[^ ^51^ ^]^ Thus, we wondered whether the correlation between DNA hemi‐methylation and nucleosome positioning is related to H3K9me2 enrichment. Interestingly, we found CG methylation only correlates with nucleosome positioning in H3K9me2‐depleted regions, while in H3K9me2‐enriched regions, both CG hemi‐ and full‐methylation are homogenous across nucleosomal and linker regions (Figure S4A, Supporting Information). This further suggests that the high methylation frequencies in nucleosome‐occupied regions are not promoted by nucleosomes. In comparison to CG, symmetric CWG and CWWG methylation are anti‐correlated with nucleosome positioning no matter H3K9me2 enrichment is low or high (Figure S4A, Supporting Information).

Since H3K9me2 has a dramatic influence on the methylation pattern of CG methylation over well‐positioned nucleosomes, we wondered whether the correlation between methylation frequencies and nucleosome positioning is affected by other epigenetic marks. To achieve this goal, we analyzed a set of ChIP‐seq data, including RNAPII, H3K4me1, H3K9ac, H3K9me2, H3K27ac, and H3K27me3.^[^ ^4^ ^]^ We calculated the Pearson correlation coefficient between methylation frequencies and nucleosome occupancy near well‐positioned nucleosomes in regions depleted or enriched by each epigenetic mark (Figure 4E). As we expected, the correlation between hemi‐/full‐methylation and nucleosome occupancy in H3K9me2‐enriched regions is much lower than that in H3K9me2‐depleted regions (Figure 4E). By contrast, the RNAPII‐enriched regions exhibit high correlation between methylation and nucleosome occupancy (Figure 4E), suggesting that gene expression may promote the co‐localization of CG methylation and nucleosomes. Additionally, in H3K27me3‐enriched regions, CG hemi‐methylation is better co‐localized with nucleosomes than in H3K27me3‐depleted regions, but this phenomenon is not observed at fully methylated CG dyads (Figure 3E). For CWG and CWWG methylation, the Pearson correlation coefficient between methylation and nucleosome occupancy is influenced by H3K4me1 and H3K27me3 (Figure S4B,C, Supporting Information). In both H3K4me1 and H3K27me3 enriched regions, the hemi‐/full‐methylation is hindered by nucleosomes, while in regions depleted with these two markers, hemi‐methylation positively correlates with nucleosome positioning.

To further investigate the causal relationship between maintenance methylation and nucleosome positioning, we examined the hemi‐ and full‐methylation patterns in a *ddm1* mutant line. Previous work suggests that DDM1 facilitates methylation in nucleosome‐occupied regions by remodeling nucleosomes.^[^ ^51^ ^]^ Our data showed that DDM1 deletion led to a much greater change in full‐ than in hemi‐methylation across all palindromic contexts (Figure 4F). Moreover, we observed an increase in hemi‐methylation levels at CG dyads, suggesting that DDM1 is required for efficient maintenance of methylation by DNA methyltransferases. We then examined how DDM1 influences methylation patterns near well‐positioned nucleosomes. Our data showed that the methylation patterns of CWG and CWWG contexts were not affected by DDM1 deletion. For CG context, in H3K9me2‐depleted regions, methylation frequencies at well‐positioned nucleosomes remained higher than in nearby linker regions (Figure 4G). Although the methylation pattern was not altered by DDM1 deletion, the full‐methylation frequency was significantly reduced. This suggests that in H3K9me2‐depleted regions, nucleosomes impair maintenance methylation, but the overall correlation pattern is determined by other factors. By contrast, in H3K9me2‐enriched regions, the deletion of DDM1 decreased the relative CG full‐methylation level near the nucleosome center (Figure 4G). This indicates that in H3K9me2‐enriched regions, nucleosome positioning affects methylation correlation patterns.

In summary, our dyad‐resolution methylation data reveal that, on the single‐nucleosome scale, both CG full‐ and hemi‐methylation frequencies remain higher in nucleosome‐occupied regions than in nucleosome‐depleted regions. In contrast, CWG and CWWG full‐methylation are more enriched in nucleosome‐depleted linker regions. These correlation patterns are associated with specific epigenetic marks, suggesting that the chromatin state may influence or be influenced by the deposition of DNA methylation. Notably, the ratios of full‐ to hemi‐methylation for all DNA contexts are higher in linker regions than nucleosomal regions, indicating that nucleosomes may generally impede maintenance methylation in *Arabidopsis*. This assumption is further supported by the methylation changes in the *ddm1* mutant.

### 2.5 Methyl Groups of Neighboring Hemi‐Methylated Dyads Tend to Occur on the Same Strand

**Figure 5:**
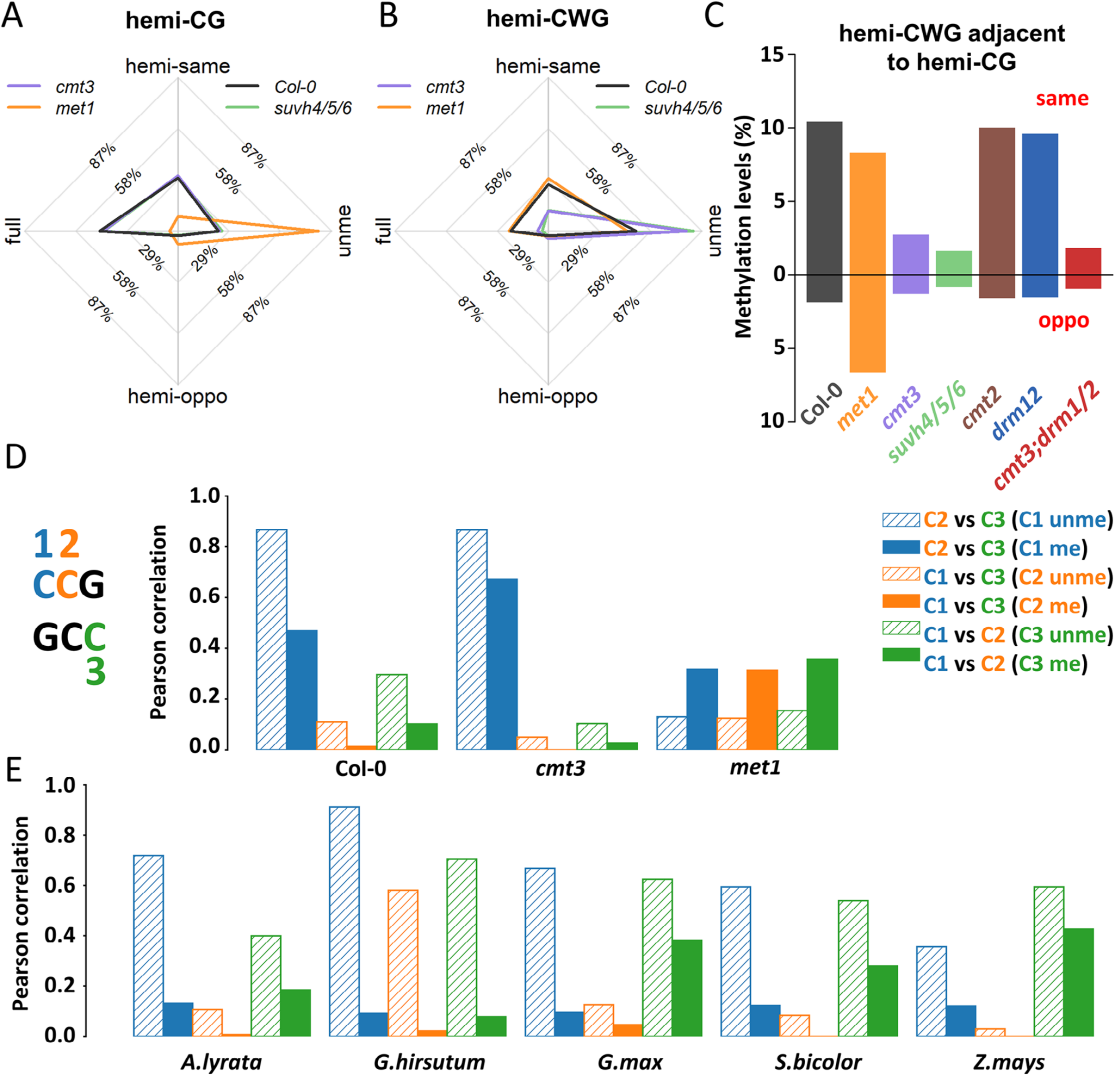
Neighboring hemi‐methylated dyads tend to have methyl groups on the same DNA strand. A,B) The methylation frequency of CG (A) or CWG (B) dyads most proximal to hemi‐methylated CG (A) or CWG (B) dyads, respectively. Hemi‐same, hemi‐oppo, full, and unme represent that the proximal dyads are hemi‐methylated on the same or opposite strand, fully‐methylated, or unmethylated, respectively. C) The frequency of hemi‐methylated CWG dyads most proximal to hemi‐methylated CG dyads. D) The Pearson correlation coefficient between cytosines in CSG dyads. The methylation status of one cytosine is fixed, and the Pearson correlation coefficient of methylation status between the other two cytosines were calculated. Unme and me represent that the fixed cytosines are unmethylated or methylated, respectively. For example, “C2 versus C3 (C1 unme)” represents the correlation between C2 and C3 when C1 is unmethylated. E) The same type of plot as (D), but the dyad‐resolved methylation information is extracted from BS‐seq datasets from *A. lyrata*, *G. hirsutum*, *G. max*, *S. bicolor*, and *Z. mays* using iSA.

Although MET1 and CMT3 can maintain symmetric methylation at CG and CWG dyads, a substantial number of hemi‐methylated dyads were still detected in *Arabidopsis*. As discussed earlier, nucleosomes assist in preserving CWG hemi‐methylation by blocking the maintenance methylation. However, the main source of hemi‐methylation remains unclear. Previous studies indicate that DNA replication plays a key role in generating hemi‐methylated CG dyads.^[^ ^2^, ^38^, ^55^ ^]^ Based on this, we hypothesized that DNA replication may be among the determinants for CG and CWG hemi‐methylation in *Arabidopsis*. To test this hypothesis, we investigated the strand specificity of neighboring methylated cytosines, as methyl groups of hemi‐methylated dyads generated during DNA replication are predominantly located on the template strands. Using paired methylation information of two DNA strands from hpBS‐seq reads, we found that hemi‐methylated dyads, irrespective of their sequence context (CG, CWG, or CHH), tend to have 5mC on the same strand of double‐stranded DNA fragments (**Figure** **5**A–C; Figure S5A, Supporting Information), suggesting that these context‐independent and same‐strand hemi‐methylated dyads may be remnants from the DNA replication process instead of *de novo* methylation. This phenomenon is also observed in most mutant lines (Figure 5A–C; Figure S5A, Supporting Information), indicating that it is not driven by the specific activity of any particular methyltransferase.

To quantitatively assess strand specificity across different DNA contexts, we analyzed methylation at the CSG dyad (S = C/G), which nests a CG dyad, with two consecutive cytosines on one strand, and a third cytosine on the other strand (Figure 5D). The three cytosines in CSG dyads were designated as C1, C2, and C3, respectively (Figure 5D). Because the correlation of methylation at two cytosines may be affected by the third cytosine, we clustered CSG sites into two groups based on the methylation status of one cytosine, and then calculated the methylation correlation for the other two cytosines (Figure 5D). For the CG dyads (C2 and C3) within CSG dyads, the methylation frequencies were highly correlated, particularly when C1 was unmethylated (Figure 5D; Figure S5B,C, Supporting Information). For C1 and C3, the correlation was positive only when C2 was unmethylated. Surprisingly, the consecutive cytosines (C1 and C2) also showed a positive correlation, which was even stronger when C3 was unmethylated. The correlation between C1 and C2 was consistently higher than that between C1 and C3, even though C2 and C3 are primarily methylated by the same enzyme, MET1. We hypothesized that this difference arises because co‐methylated C1 and C2 remain on the same DNA strand after DNA replication, while co‐methylated C1 and C3 are separated during DNA replication. Therefore, these results suggest that the strand‐specific hemi‐methylation pattern may be established by DNA replication.

To investigate whether DNA methyltransferases influence strand‐specific DNA methylation, we analyzed CSG dyad methylation in mutant lines (Figure 5D; Figure S5B,C, Supporting Information). As expected, the loss of MET1 eliminated the strong correlation between C2 and C3 in the CG dyad, and increased the correlation between C1 and C3 in the CSG dyad. Surprisingly, despite C1 and C3 being methylated by CMT3, the correlation between C1 and C2 remained stronger than that between C1 and C3 in the *met1* mutant (Figure 5D). This suggests that CMT3 is inefficient at maintaining symmetric CSG methylation after DNA replication. The deletion of CMT3 and SUVH4/5/6 greatly reduced the correlation between C1 and the other cytosines (Figure 5D; Figure S5B,C, Supporting Information). Without interference from C1, the correlation between C2 and C3 increased (Figure 5D). As CMT2 and DRM1/2 exhibit low activity at cytosines in the CCG context, no significant changes were observed in *cmt2* and *drm1/2* mutants (Figure S5B, Supporting Information). These results indicate that strand‐specific hemi‐methylation arises from DNA replication and persists when symmetric methylation is not maintained in a timely manner. Since our experiments only took a snapshot of the methylomes from cell populations, these hemi‐methylated dyads may still undergo maintenance methylation later on.

Given the ubiquitous presence of hemi‐methylated CG and CHG dyads in plants, we investigated whether similar methylation patterns occur at CSG dyads in other plant species. Using dyad‐resolution DNA methylation data retrieved from WGBS datasets via iSA for *A. lyrata*, *G. max, S. bicolor, Z. mays, and G. hirsutum* (Figure 5E; Figure S5D,E, Supporting Information), we observed similar correlations between cytosines in CSG dyads across these species as in *A. thaliana*.

Overall, our findings suggest that the homeostasis of hemi‐methylation in plants can be attributed to multiple factors, including nucleosome positioning as we described in last section, and also DNA replication, possibly due to relatively lagged methylation maintenance process, giving rise to a time window after the passage of DNA replication forks for strand‐specific hemi‐methylation to be observed.^[^ ^55^ ^]^

## 3 Discussion

Standard WGBS cannot distinguish between fully methylated and hemi‐methylated dyads, since two DNA strands from the same molecule cannot re‐anneal due to conversions of cytosines into uracils. As a result, each read in WGBS data represents the methylation status of only one strand of a double‐strand DNA molecule. To acquire dyad‐resolution methylome from WGBS, we have developed an in silico method called iSA, albeit with low pairing efficiency and requirement for very high sequencing depth.^[^ ^35^, ^43^ ^]^ In contrast, hpBS‐seq employs a hairpin adapter to covalently link the two strands of the same DNA molecule, preserving dyad‐resolution methylation information within single sequencing reads.^[^ ^39^ ^]^ However, the library preparation efficiency is limited by the low ligation efficiency of hairpin adapters and DNA damage caused by bisulfite conversion. Thus, hpBS‐seq requires a large amount of input DNA for adequate library preparation. Single‐molecule long‐read sequencing technologies, such as those based on PacBio or Nanopore platforms, can also be used to detect strand‐specific hemi‐methylation. The long reads produced by these platforms enable the mapping of repetitive sequences, providing more complete methylomes than WGBS and hpBS‐seq.^[^ ^56^, ^57^ ^]^ However, specialized data processing algorithms or experiment procedures need to be developed to call strand‐specific methylation information.^[^ ^57^, ^58^ ^]^ Additionally, the costs for PacBio and Nanopore sequencing are higher compared to regular short‐read sequencing.^[^ ^57^ ^]^ For these reasons, hpBS‐seq is currently still the gold standard in detecting hemi‐methylation.

Our data revealed that in *Arabidopsis*, methylation maintenance efficiencies are generally higher in euchromatic regions than in heterochromatic regions when total methylation frequencies are similar (Figure 3A,B). We propose that this difference may relate to the concentration of the substrate. In euchromatic regions, the overall methylation frequency is relatively low, resulting in a high enzyme‐to‐substrate ratio. By contrast, heterochromatic regions are highly methylated, leading to a low enzyme‐to‐substrate ratio. When the substrate concentration greatly exceeds that of the enzymes, the reaction velocity becomes limited by enzyme availability. Thus, to achieve high maintenance efficiency, heterochromatic regions would need to recruit more enzymes or enhance enzyme catalytic activity, for example via H3K9me2. Even so, heterochromatic regions can still maintain high methylation levels across multiple cell cycles. The maintenance methylation efficiency is defined by the methylation rate. For instance, if two populations of cells take 10 min and 10 h, respectively, to fully methylate all hemi‐methylated dyads, the first population will have a higher maintenance efficiency than the second population, even though both populations ultimately reach the same methylation level before the next round of replication. Therefore, even in regions with low maintenance efficiency, DNA methylation can be stably maintained after multiple rounds of cell division, given that maintenance methylation is completed before the onset of the next cell cycle. The cells will gradually lose DNA methylation only when the maintenance methylation is not completed prior to the next cell cycle. To analyze maintenance rate in a more quantitative manner, a time‐course study could be conducted using synchronized cells. In addition to maintenance methylation, the maintenance efficiency we defined is also influenced by *de novo* methylation and ROS1‐mediated demethylation.^[^ ^2^, ^45^, ^59^, ^60^ ^]^ Our analysis of the strand‐specificity of adjacent hemi‐methylated dyads revealed a small subset of CG and CWG dyads exhibiting 5mC on opposite strands, indicating that at least one *de novo* methylation or demethylation event occurred.

We and other groups have identified strand‐specific hemi‐methylation in mammalian cells.^[^ ^35^, ^36^, ^61^, ^62^ ^]^ However, in *Arabidopsis*, we observed strand‐specific hemi‐methylation only at the single‐molecule level, but not in cell populations. We propose two possible explanations for this. First, strand‐specific hemi‐methylation may be obscured by the background hemi‐methylation. Second, *Arabidopsis* may lack factors that mediate strand‐specific methylation. In mammalian cells, strand‐specific hemi‐methylation has been observed near CTCF motifs, potentially due to the directional binding of CTCF and the unique chromatin structure at Topologically associating domain boundaries.^[^ ^35^, ^36^ ^]^ In contrast, there are no CTCF homologs in the *Arabidopsis* genome. However, we cannot rule out the possibility that strand‐specific hemi‐methylation may facilitate or interfere with the binding of TFs in *Arabidopsis*. The effect of full‐ and hemi‐methylation on TF binding could be further evaluated via in vitro assays, such as Systematic Evolution of Ligands by Exponential Enrichment (SELEX) and Electrophoretic Mobility Shift Assay (EMSA).^[^ ^63^, ^64^ ^]^ In mammals, abnormal hemi‐methylation frequencies are often associated with diseases.^[^ ^61^, ^65^ ^]^ Hemi‐methylation may also serve as a potential biomarker for monitoring epigenetic alterations in plants.

The relationship between DNA methylation and nucleosome positioning has been studied for decades, but controversies still exist on this topic due to the over‐simplified conditions of in vitro experiments and the complicated intracellular environments.^[^ ^47^, ^48^, ^49^, ^50^, ^52^, ^53^, ^54^, ^66^ ^]^ In our study, by resolving methylation at dyad resolution in Col‐0 and *ddm1* mutant lines, we confirmed that nucleosomes inhibit the maintenance of methylation at hemi‐methylated dyads. However, we still observed a strong positive correlation between nucleosome occupancy and CG methylation. This correlation may be due to the intrinsic sequence preferences of nucleosomes.^[^ ^67^, ^68^ ^]^ Previous studies revealed that both DNA methylation and nucleosome positioning influence and are influenced by transcription.^[^ ^1^, ^4^, ^36^, ^51^, ^63^, ^69^, ^70^, ^71^, ^72^, ^73^, ^74^, ^75^, ^76^, ^77^, ^78^, ^79^ ^]^ Our data suggest that symmetric methylation is more efficiently maintained in highly transcribed regions than in poorly expressed genes. Therefore, the observed correlations would be shaped by the interplay among DNA methylation, nucleosome positioning, and transcription. To further dissect the role of transcription in maintenance methylation, hpBS‐seq could be performed in cells treated with transcription inhibitors to investigate the changes of full‐ and hemi‐methylation upon the halt of transcription.

**Figure 6:**
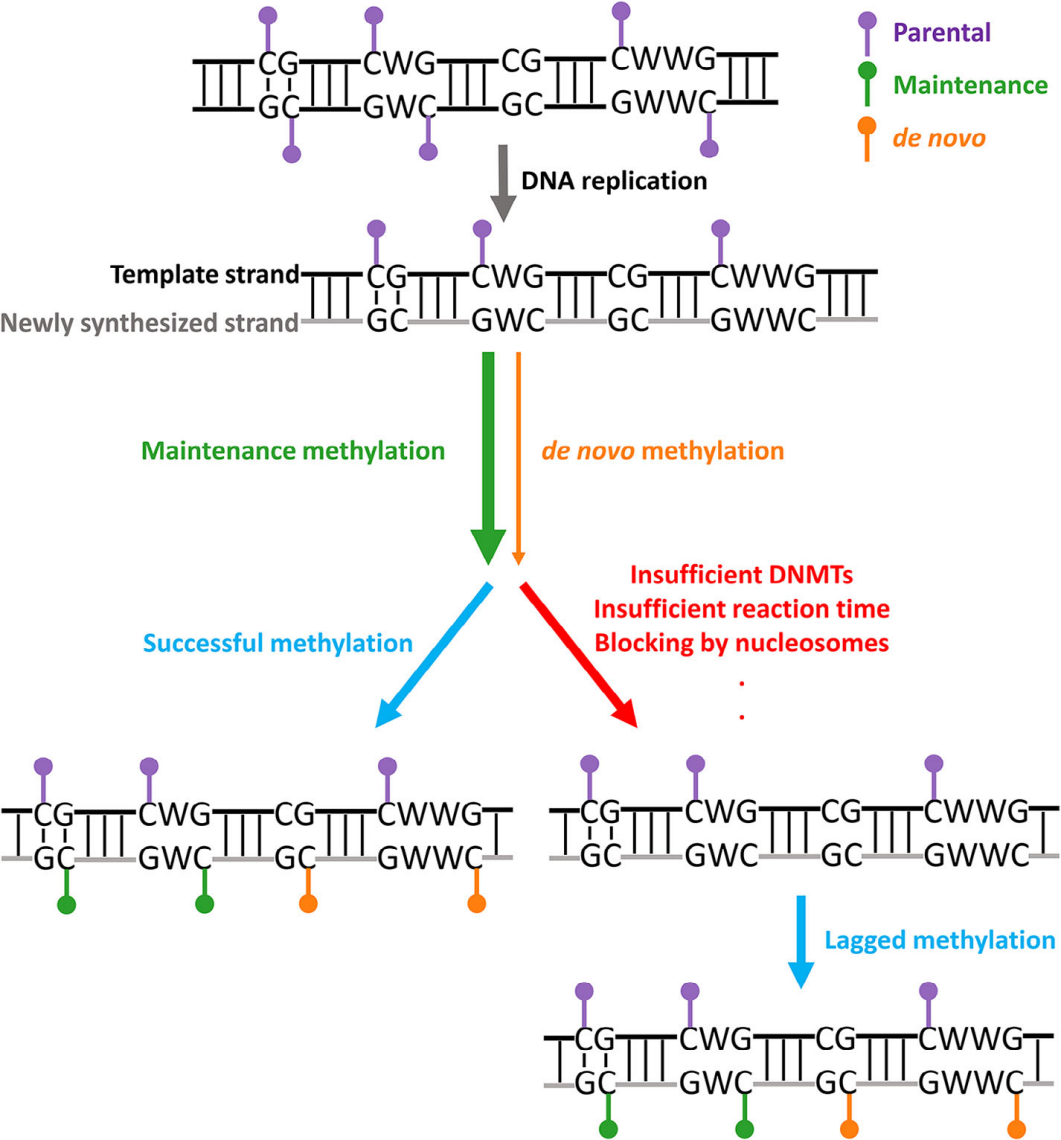
Homeostasis of DNA hemi‐methylation during DNA replication and maintenance methylation. During DNA replication, unmethylated cytosines were incorporated into newly synthesized daughter DNA strands. Maintenance methylation efficiently converts these hemi‐methylated CG and CWG dyads into fully methylated ones, while *de novo* methylation adds methyl groups to all types of unmethylated cytosines. However, the hemi‐methylated dyads may not be converted in a timely manner, resulting in time windows when strand‐specific hemi‐methylation can be observed. These hemi‐methylated dyads may still undergo maintenance methylation later.

Taken together, we build a model for the homeostasis of hemi‐methylation in *Arabidopsis* (**Figure** **6**). During DNA replication, the newly synthesized DNA strand is methylation‐free, resulting in DNA molecules only with hemi‐methylated or unmethylated cytosine dyads. The majority of hemi‐methylated CG and CWG dyads were specifically converted to symmetric full‐methylation via maintenance methylation, while a small amount of unmethylated cytosines are methylated via *de novo* methylation. Although maintenance methylation is generally highly efficient, some hemi‐methylated dyads persist due to various factors (Figure 6), allowing the acquisition of strand‐specific methylation patterns on single DNA molecules by hpBS‐seq.

## 4 Conclusion

The DNA methylome is an equilibrium composed of full‐methylation, hemi‐methylation, and un‐methylation statuses of cytosine dyads. It is influenced not only by processes depositing methyl groups onto DNA, like maintenance methylation and *de novo* methylation, but also by processes leading to loss or dilution of methyl groups, such as demethylation and DNA replication. The consequences of these mutually counteractive processes converge at hemi‐methylation, making its content within a DNA methylome highly dynamic and informative. We reveal efficient maintenance methylation for both CG and CWG dyads at H3K9me2‐depleted regions, consistent with indirect or direct recruitment of MET1 or CMT3 to hemi‐methylated dyads. At H3K9me2‐enriched regions, the CG and CWG maintenance methylation is less efficient, possibly due to the enzymes being rate‐limiting, giving rise to higher‐than‐expected levels of hemi‐methylation. Particularly, the crosstalk between H3K9me2 and CWG methylation serves as a maintenance methylation mechanism distinct from the one canonically defined by the use of hemi‐methylated dyads as templates. This may help explain the distinct kinetics of CWG maintenance methylation in H3K9me2‐enriched or ‐depleted regions.^[^ ^10^, ^18^, ^19^, ^22^, ^25^, ^26^ ^]^ The CHH methylation is predominantly carried out through *de novo* methylation. Although we observed a small fraction of fully‐methylated CWWG dyads, our modeling suggests two separate *de novo* methylation events being the main causes. Therefore, our analysis suggests that the distinction between maintenance methylation and *de novo* methylation does not simply lie within substrate cytosine contexts or methylation statuses, but could be highly chromatin context‐specific.

## 5 Experimental Section

### Plant Culture and DNA Extraction

All WT and mutant lines used in this study were of the Columbia ecotype and could be bought from the Arabidopsis Biological Resource Center (https://abrc.osu.edu). Genotypes and IDs of these lines were listed in Table S2 (Supporting Information). Plants were grown at 23 °C under a 16 h light / 8 h dark photo‐period. The leaves of 3 to 4‐week‐old plants were collected, frozen in liquid nitrogen, and ground using a mortar and pestle. Genomic DNA was then extracted from ground samples using the DNeasy Plant Mini Kit (69104, QIAGEN).

### Hairpin Bisulfite Sequencing (hpBS‐seq)

The hpBS‐seq was performed as described in a previous work.^[^ ^36^ ^]^ 3–10 µg of genomic DNA was sonicated to 100–200 bp fragments using a Bioruptor. 1 µg of sheared DNA, 1.5 µL of NEB End Repair Enzyme Mix (E6050L, NEB), and 3 µL of 10x NEB End Repair Reaction Buffer were mixed in a 30 µL reaction and incubated at 20–25 °C for 30 min. Repaired DNA was then cleaned‐up by AMPure beads (18% PEG, 1:1.8) and eluted in 25 µL of UltraPure Water (10977023, Thermo Fisher). 1 µL of *Taq* DNA Polymerase (EP0402, Thermo Fisher), 3 µL of 10x A‐tailing mix (100 µL of 10x ThermoPol Reaction Buffer (B9004S, NEB), and 1 µL of 100 mm dATP) were added to the eluted DNA, followed by incubation at 37 °C for 30 min. DNA was cleaned‐up by AMPure beads (18% PEG, 1:1.8) and eluted in 25 µL of water again. 3 µL of 10x T4 DNA Ligase Buffer (Thermo Fisher), 0.2 µL of 9 µm hairpin adapter (/5Phos/CGCCGGCGGCAAG/iBiodT/GAAGCC GCCGGCGT), 0.8 µL of Illumina TruSeq DNA PCR‐Free adapter (20015960, Illumina), and 0.8 µL of T4 DNA Ligase (EL0012, Thermo Fisher) were mixed with the eluted DNA, followed by a 2‐h incubation at 20–25 °C. DNA was then cleaned‐up by AMPure beads (18% PEG, 1:1.4). The DNA sample was mixed with Dynabeads Streptavidin C1 beads (65002, Thermo Fisher) and rotated for 30 min at 20–25 °C to enrich DNA ligated with the biotinylated adapter. The beads were then sequentially washed by 500 µL of 1x BW Buffer, 200 µL of 150 mm NaOH with 0.01% Tween‐20, and 200 µL of 10 mm Tris‐HCl, pH 8.0. DNA was eluted by incubating with 95% formamide and 10 mm EDTA pH 8.2 at 95 °C for 3 min. Eluted DNA was precipitated by ethanol, dissolved in water, and bisulfite‐converted with the EZ DNA Methylation‐Lightning Kit (D5030, Zymo). Converted DNA was then amplified using HiFi HotStart Uracil+ DNA Polymerase (KK2801, Roche) with the following PCR cycling condition: 95 °C 3 min for 1 cycle; 98 °C 20 s, 60 °C 15 s, and 72 °C 30 s for 11–13 cycles; and 72 °C 1 min for 1 cycle. The PCR product was cleaned‐up by AMPure beads (18% PEG, 1:1) and eluted in water. The final amount of DNA should be ≈50 ng. The libraries were sequenced using an Illumina HiSeq2500 pair‐end sequencing platform.

### Statistical Analysis—hpBS‐seq Data Preprocessing

The 50 bp paired‐end reads were initially trimmed using Trimmomatic (0.39) to eliminate any remaining hairpin linker sequence and adapter sequence.^[^ ^80^ ^]^ Subsequently, the mate 1 and mate 2 reads were individually aligned to the Arabidopsis genome (TAIR10) using Bismark (0.23.0) with bowtie2 (2.4.5).^[^ ^81^, ^82^ ^]^ The alignment score was controlled by applying the bowtie2 option –score‐min L,0,−0.4, and mapping uniqueness was ensured by selecting alignments with a mapping quality MAPQ > 10 using Samtools (1.16.1).^[^ ^83^ ^]^ Duplicate reads were then removed through Bismark (0.23.0). The methylation status of each cytosine on the forward and reverse reads was extracted and saved in two bed files, respectively.^[^ ^84^ ^]^ Cytosines from each read pair were labeled by a unique index. The cytosines in the two bed files were then paired based on their genomic position and index. The methylation status of the paired cytosines was used to determine the methylation status at the CG, CWG, and CWWG dyads. Each dyad could have one of the four statuses: 1) unmethylated, 2) only Watson strand methylated (hemi‐methylation on Watson strand), 3) only Crick strand methylated (hemi‐methylation on Crick strand), and 4) both strands methylated (full‐methylation). The counts of these statuses were then used to calculate methylation frequencies at each resolution. A two‐sided student *t*‐test was performed to evaluate the changes of methylation in mutant lines via *scipy.stats.ttest_rel* function in Python. Significance was defined as *P* ≤ 0.05. The bisulfite conversion rate of each library was calculated according to the unmethylated cytosines on the lambda sequence. The code is available at https://github.com/HengyeChen/Ath_hpBS_seq.

### Statistical Analysis—iSA

iSA was performed as described before.^[^ ^43^ ^]^ In brief, paired‐end reads from bisulfite sequencing were aligned to either the Watson or Crick strands using Bismark. Since the Watson and Crick strands from the same DNA molecule before bisulfite conversion exhibit the same genomic coordinates, an iterative search was then performed between alignments on Watson and Crick strands using Samtools and Bedtools based on their genomic coordinates. Watson and Crick strands that share the same genomic coordinates were retained for downstream analysis. Because random fragmentation during bisulfite conversion could also generate reads with the same coordinates, the pairing accuracy was evaluated by comparing the number of same‐ends pairs and the estimated random pairing reads. From a WGBS dataset with sufficient genome coverage, a 10—100‐fold enrichment of same‐ends over random pairing was expected to be observed.

### Statistical Analysis—DMR Calling

Two‐sided student *t*‐test and Mann–Whitney U test were performed to calculate *P*‐values for full‐ or hemi‐methylation frequencies at dyads in each 1‐kb bin via *scipy.stats.ttest_rel* and *scipy.stats.mannwhitneyu* functions in Python. Bins with FDR‐adjusted *P*‐value < 0.05 were defined as fDMRs or hDMRs. For regions completely lost methylation in the mutant lines, if these bins contain > 10 dyads, and methylation changes were > 30%, they were defined as DMRs as well.

### Statistical Analysis—Curve Fitting

Full‐ and hemi‐methylation frequencies were calculated at dyads covered by ≥ 5 reads. The methylation frequencies at each dyad were then used to calculate the average methylation frequencies in each 1 kb bin. Bins with total methylation lower than 2.5% were removed due to the high noise. bedtools (2.30.0) was used to extract bins with high and low enrichments of certain marks. The parameters of the equation were fitted by minimizing the mean square error (MSE) via *scipy.optimize.minimize* function in Python.

### Statistical Analysis—Nucleosome Calling

Raw sequencing files from dataset GSE96994 were trimmed by Trimmomatic (0.39) and aligned to the *Arabidopsis* genome using bowtie2 (2.4.5).^[^ ^51^, ^80^, ^81^ ^]^ The data were then processed by iNPS for nucleosome calling.^[^ ^85^ ^]^ Nucleosomes with both ‐log10(Pvalue_of_peak) and ‐log10(Pvalue_of_valley) larger than 0.5 were used for the subsequent analysis.

### Statistical Analysis—ChIP‐seq Data Analysis

Processed ChIP‐seq BigWig files of dataset GSE183957 were downloaded from GEO.^[^ ^4^ ^]^ A blacklist was generated from the input file to remove regions with coverage above two‐fold of the mean coverage. The average enrichment score was calculated in 1 kb bins via *bigWigToBedGraph* and bedtools.^[^ ^84^ ^]^ For H3K9me2, bins with enrichment ≤ 0.1 or ≥ 5 were defined as low or high enrichment regions, respectively. For other marks, bins with enrichment ≥ 2 were defined as high enrichment regions.

### Statistical Analysis—CSG Methylation Correlation Analysis

Methylation status at CSG dyads was extracted and presented as 0 or 1 for each cytosine, where 0 and 1 represent unmethylated and methylated cytosine, respectively. The Pearson correlation coefficient was then calculated for each cytosine pair (C1‐C2, C2‐C3, or C1‐C3) when the other cytosine was unmethylated or methylated.

## Conflict of Interest

The authors declare no conflict of interest.

## Supporting information



Supporting Information

## Data Availability

The data that support the findings of this study are openly available in GSA at https://ngdc.cncb.ac.cn/gsa/browse/CRA023911.
