# Supplementary material for: Homeostasis of DNA Hemi‐Methylation in Arabidopsis through Methylation Maintenance, DNA Replication, and Nucleosome Positioning Mechanisms
Source: Adv Sci (Weinh). 2025 Jul 11;12(38):e05808. doi: 10.1002/advs.202505808 (PMC12520501; doi:10.1002/advs.202505808)
Supplement: Supplementary file 1 — Supporting Information [file ADVS-12-e05808-s001.pdf]

ADVANCED SCIENCE
Open Access

Supporting Information

for *Adv. Sci.*, DOI 10.1002/advs.202505808

Homeostasis of DNA Hemi-Methylation in *Arabidopsis* through Methylation Maintenance, DNA Replication, and Nucleosome Positioning Mechanisms

*Hengye Chen and Chenhuan Xu**

Supplementary Figure S1

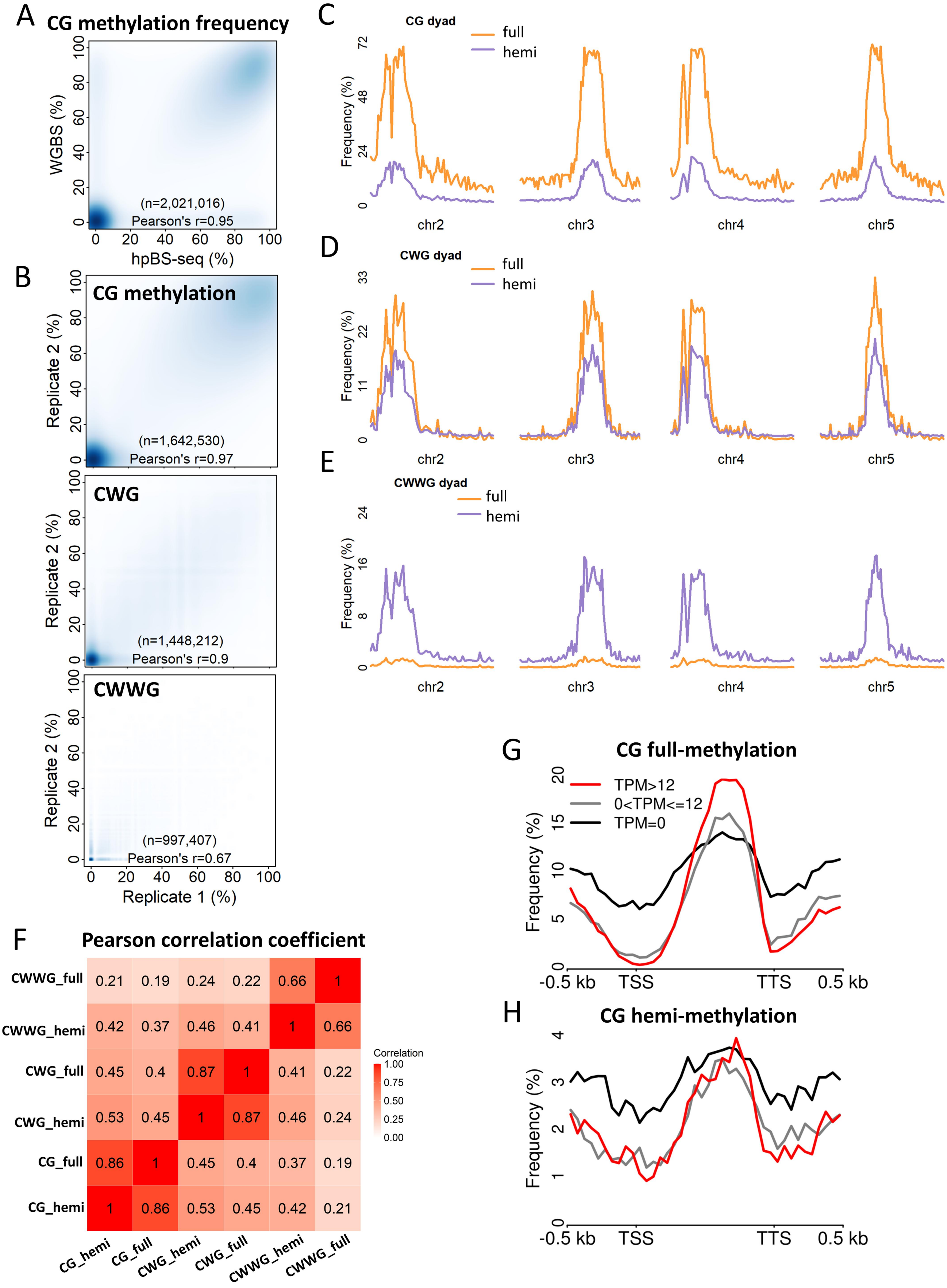

Supplementary Figure S1. DNA full- and hemi-methylation frequency at different DNA contexts in *Arabidopsis* thaliana and other plant species.

A) Correlation of CG methylation frequencies between WGBS and hpBS-seq in Col-0. B) Correlation of CG, CWG, or CWWG dyad methylation frequencies between hpBS-seq replicates in Col-0. C-E) Hemi- and full-methylation frequency of CG, CWG, or CWWG dyads on Chromosomes 2-5 at 300-kb resolution. F) Pearson correlation coefficient between hemi- and full-methylation frequency of CG, CWG, and CWWG dyads at 1-kb resolution. G and H) CG full- (G) and hemi-methylation (H) frequencies on highly (red), moderately (grey), and poorly (black) transcribed genes. TPM: transcript per million reads.

Supplementary Figure 2

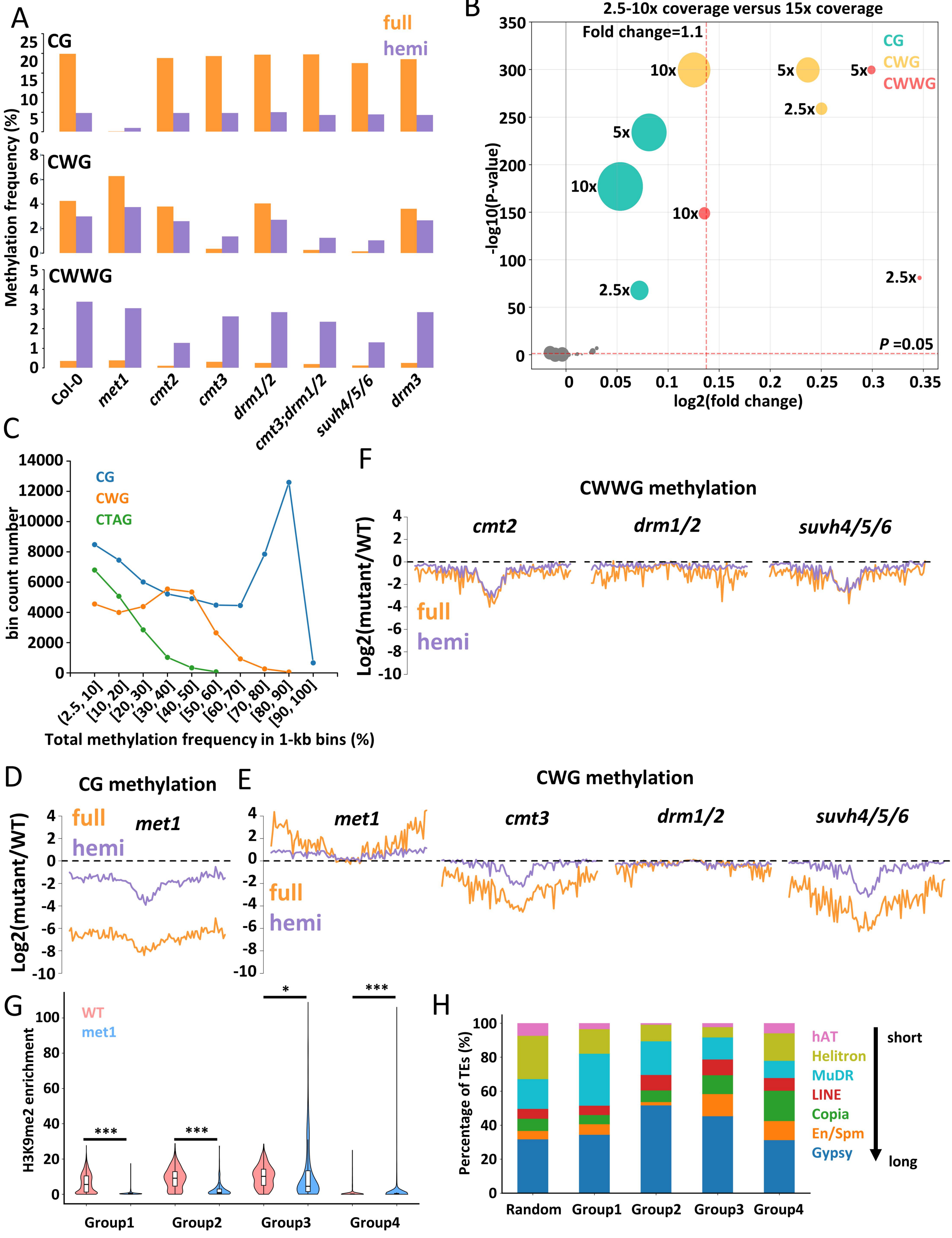

Supplementary Figure. S2. Homeostasis of full- and hemi-methylation is co-regulated by multiple enzymes.

A) Genome-wide full- and hemi-methylation levels in Col-0 and mutant lines. B) Comparison between 2.5-10x coverage and 15x coverage data from dyad to 300-kb resolution. 1-kb to 300-kb resolution are indicated by the grey dots, while dyad resolution results are indicated by green (CG), yellow (CWG), and red (CWWG) colors. The horizontal and vertical red lines represent *P*-value=0.05 and fold change=1.1, respectively. *P*-value above 1E+300 was set as 1E+300. The coverages are labelled on the side of the bubbles for dyad-resolution data. C) The number of 1-kb bins with different methylation levels. D-F) The changes of CpG (D), CWG (E), and CWWG (F) hemi- and full-methylation levels at Chromosome 1 in mutant strains. The fold change of methylation was plotted in log scale. The resolution is 300-kb. In each plot, more than 30% of bins exhibit significant fold-change in full-methylation (adjusted $P<0.05$). G) H3K9me2 enrichment in Groups 1-4 fDMRs of met1 mutant line. H) The percentage of each TE element in Groups 1-4 fDMRs. The sum of percentages was adjusted to 100%. TEs were ordered by length from short (top) to long (bottom). Student's t test was used to calculate the significance *P*-value. *, $P < 0.05$; ***, $P < 0.001$.

Supplementary Figure S3

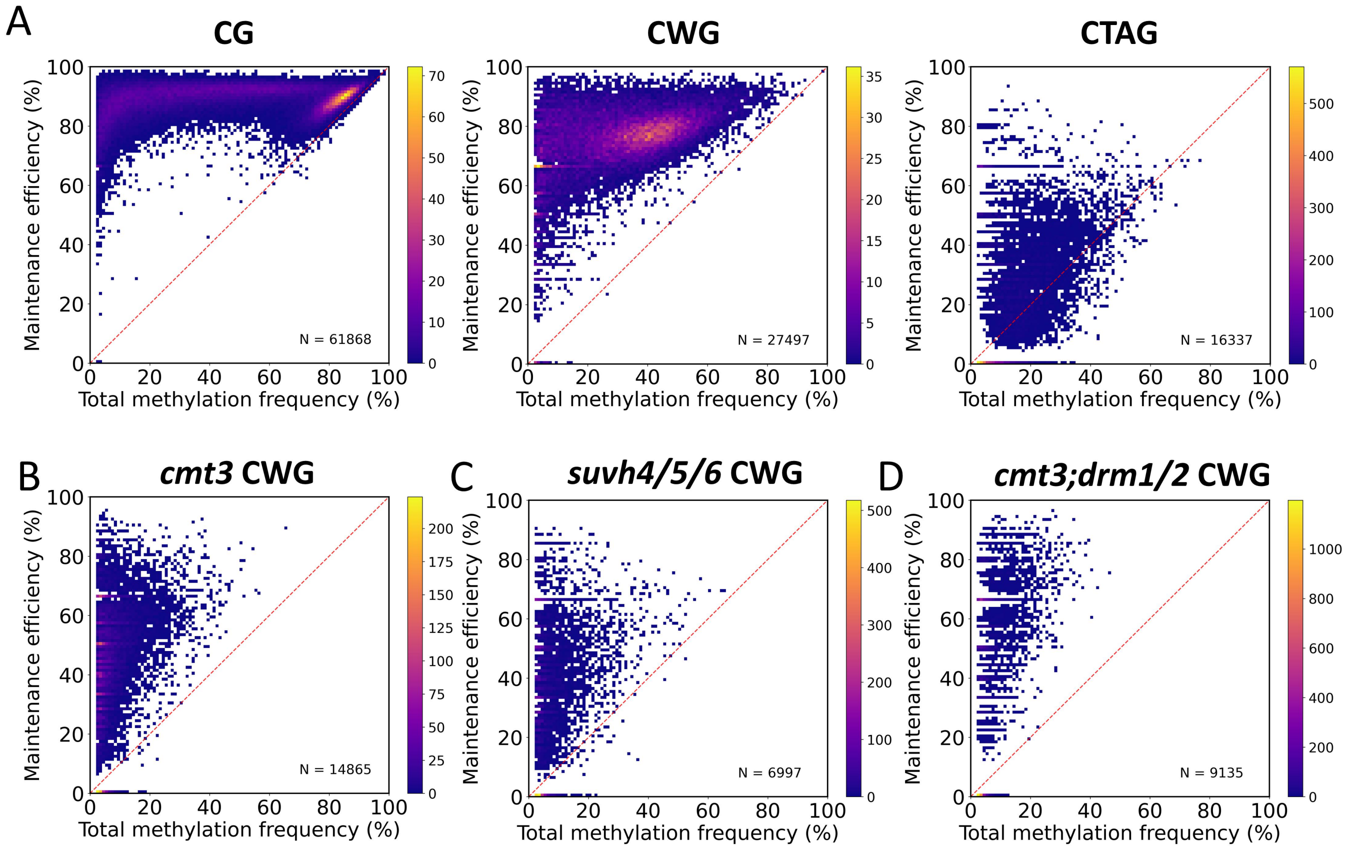

Supplementary Figure S3. Differential maintenance methylation efficiency at H3K9me2-enriched and -depleted regions.

A) The maintenance efficiency is plotted versus the total methylation in 1-kb bins for CG, CWG, and CTAG. N: the count of bins. The red line represents y=x. B-D) The relationship between methylation maintenance efficiency and total methylation frequency at CWG dyads in *cmt3* (B), *suvh4/5/6* (C), and *cmt3;drm1/2* (D) mutants.

Supplementary Figure S4

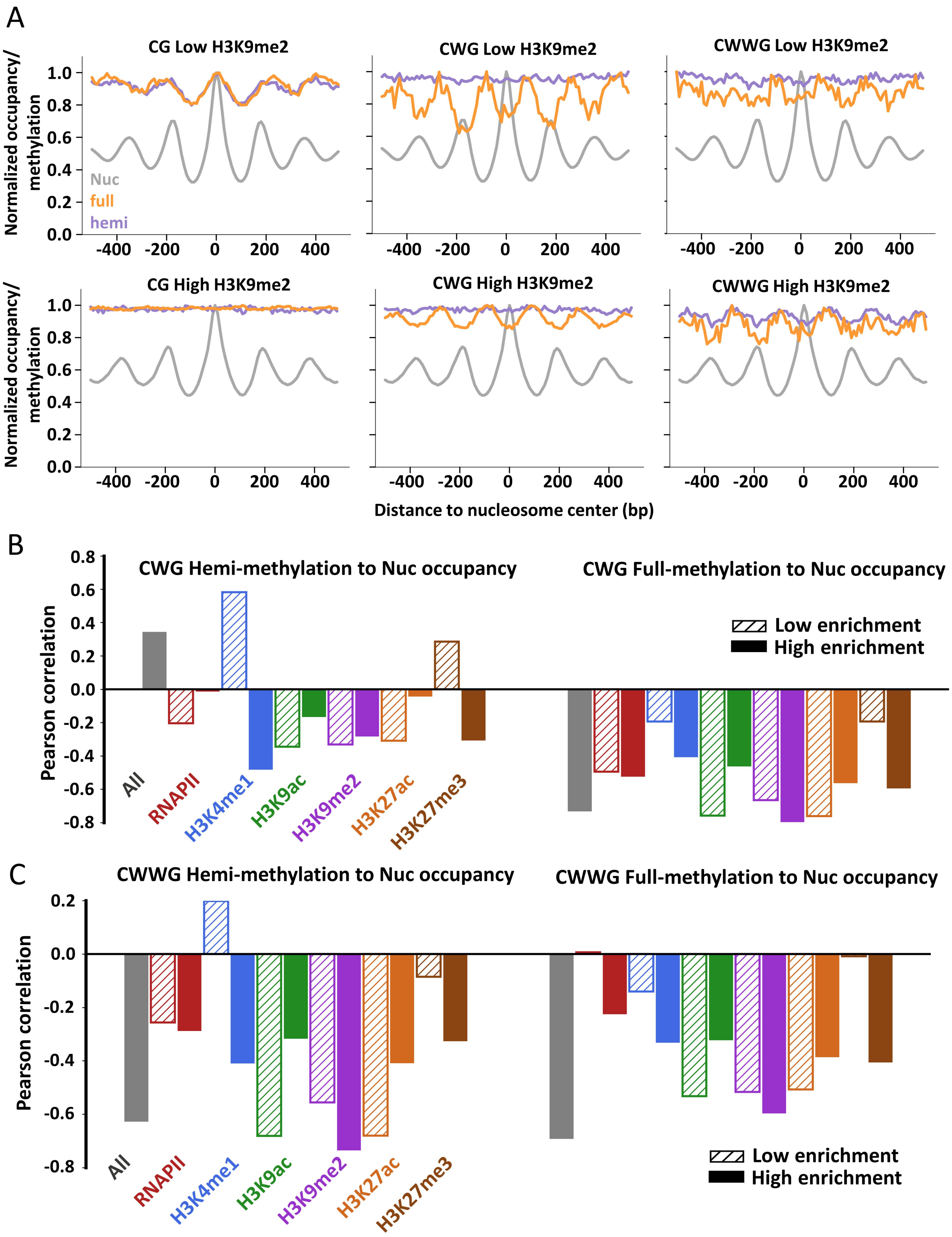

Supplementary Figure S4. CG, CWG, and CWWG methylation maintenance is interfered by nucleosomes.

A) Nucleosome occupancy level and full-methylation frequency of CG, CWG, and CWWG dyads in H3K9me2-depleted or -enriched regions. B and C) Pearson correlation coefficient between CWG (B) and CWWG (C) methylation frequency and nucleosome occupancy level near well-positioned nucleosomes in regions with different epigenetic marks. The grey bar represents the genome-wide correlation. Bars filled by hatches or solid colors represent correlation in regions with low or high enrichment of target marks, respectively.

Supplementary Figure S5

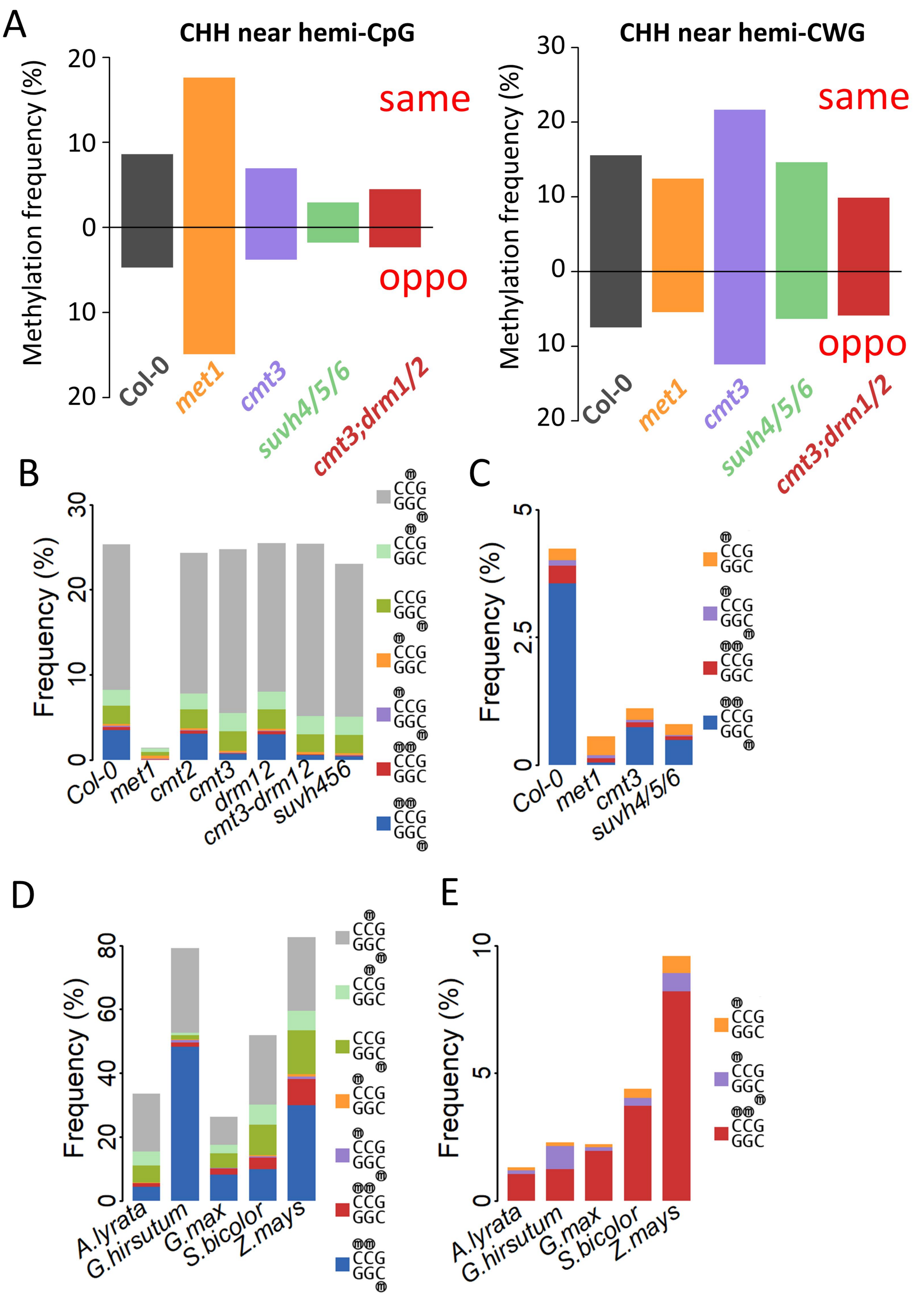

Supplementary Figure S5. Neighboring hemi-methylated dyads tend to have methyl groups on the same DNA strand.

A) The methylation frequency of CHH dyads adjacent to hemi-methylated CG or CWG dyads. Same and oppo represent that the adjacent dyads are hemi-methylated on the same or opposite strand, respectively. B) The frequency of each CSG dyad methylation status in WT and mutant lines. The small "m" near C marks the "methylated" status of this cytosine. C) Same as (B), but only CSG dyads with C1 methylated are shown in the plot. D) Same as (B), but CSG dyads from other plant species were plotted. E) The three most poorly methylated statuses in (D).

Supplementary Table S1. List of mutant lines used in this study.

name in this paper	mutant	stock number	background	references
ddm1	*ddm1-10*	SALK_093009	Col-0	Alonso et al., Science. 2003. PMID: 12893945.<br>Jordan et al. Plant Mol Biol. 2007. PMID: 17786563.
cmt2	*cmt2-3*	SALK_012874	Col-0	Alonso et al., Science. 2003. PMID: 12893945.<br>Stroud et al. Nat Struct Mol Biol. 2014. PMID: 243362
drm3	*drm3-1*	SALK_136439	Col-0	Alonso et al., Science. 2003. PMID: 12893945.<br>Stroud et al., Cell. 2015. PMID: 23313553
cmt3;drm1/2	*cmt3-11t;drm1-2;drm2-2*	CS16384	Col-0	Zhang et al., Cell. 2006. PMID: 16949657.<br>Chan et al., PLoS Genet. 2006. PMID: 16741558
drm1/2	*drm1-2;drm2-2*	CS16383	Col-0	Henderson et al., Genes Dev. 2008. PMID: 18559476<br>Stroud et al., Cell. 2015. PMID: 23313553
suvh4/5/6	*suvh4;suvh5;suvh6*	CS72758	Col-0	Stroud et al., Cell. 2015. PMID: 23313553<br>Li et al., PNAS. 2018. PMID: 30150382
met1	*met1-3*	CS16394	Col-0	Johnson et al., Curr Biol. 2007. PMID: 17239600<br>Stroud et al., Cell. 2015. PMID: 23313553
cmt3	*cmt3-11t*	SALK_148381	Col-0	Alonso et al., Science. 2003. PMID: 12893945.<br>Stroud et al. Nat Struct Mol Biol. 2014. PMID: 243362<br>Stroud et al., Cell. 2015. PMID: 23313553

Supplementary Table S2. Sources and accession numbers of public datasets used in this study.

Experiment	Species	ID	ID	ID	ID	ID
WGBS	Ath	SRR501624	SRR534177	SRR534193	SRR24421266	SRR24421270
WGBS	Aly	ERR575711	ERR575715			
WGBS	Ghi	SRR1586244	SRR1586245			
WGBS	Gma	SRR5044695	SRR5044696			
WGBS	Sbi	SRR2102348	SRR2102349	SRR2102350		
WGBS	Zma	GSM958913	GSM958914	GSM958915		

	target	ID	ID
ChIP-seq	Input	GSM5574964	GSM5574965
	RNAPII	GSM5574962	GSM5574963
	H3K4me1	GSM5574954	GSM5574955
	H3K9ac	GSM5574956	GSM5574957
	H3K9me2	GSM5574960	GSM5574961
	H3K27ac	GSM5574958	GSM5574959
	H3K27me3	GSM5574952	GSM5574953

	Species	ID	ID
Mnase-seq	Ath Col-0	SRR6156822	SRR6156823
RNA-seq	Ath Col-0	SRR1005385	SRR1005386
